# Systematic Optimization of Automated Phosphopeptide Enrichment for High-Sensitivity Phosphoproteomics

**DOI:** 10.1016/j.mcpro.2024.100754

**Published:** 2024-03-27

**Authors:** Patricia Bortel, Ilaria Piga, Claire Koenig, Christopher Gerner, Ana Martinez-Val, Jesper V. Olsen

**Affiliations:** 1 Faculty of Chemistry, Department of Analytical Chemistry, University of Vienna, Vienna, Austria; 2 Vienna Doctoral School in Chemistry (DoSChem), University of Vienna, Vienna, Austria; 3 Proteomics Program, Faculty of Health and Medical Sciences, Novo Nordisk Foundation Center for Protein Research, University of Copenhagen, Copenhagen, Denmark; 4 Joint Metabolome Facility, University of Vienna and Medical University of Vienna, Vienna, Austria

**Keywords:** phosphoproteomics, automation, Orbitrap Astral, Orbitrap Exploris, data independent acquisition, sequential enrichment

## Abstract

Improving coverage, robustness, and sensitivity is crucial for routine phosphoproteomics analysis by single-shot liquid chromatography-tandem mass spectrometry (LC-MS/MS) from minimal peptide inputs. Here, we systematically optimized key experimental parameters for automated on-bead phosphoproteomics sample preparation with a focus on low-input samples. Assessing the number of identified phosphopeptides, enrichment efficiency, site localization scores, and relative enrichment of multiply-phosphorylated peptides pinpointed critical variables influencing the resulting phosphoproteome. Optimizing glycolic acid concentration in the loading buffer, percentage of ammonium hydroxide in the elution buffer, peptide-to-beads ratio, binding time, sample, and loading buffer volumes allowed us to confidently identify >16,000 phosphopeptides in half-an-hour LC-MS/MS on an Orbitrap Exploris 480 using 30 μg of peptides as starting material. Furthermore, we evaluated how sequential enrichment can boost phosphoproteome coverage and showed that pooling fractions into a single LC-MS/MS analysis increased the depth. We also present an alternative phosphopeptide enrichment strategy based on stepwise addition of beads thereby boosting phosphoproteome coverage by 20%. Finally, we applied our optimized strategy to evaluate phosphoproteome depth with the Orbitrap Astral MS using a cell dilution series and were able to identify >32,000 phosphopeptides from 0.5 million HeLa cells in half-an-hour LC-MS/MS using narrow-window data-independent acquisition (nDIA).

## Highlights

• Systematic optimization of parameters for automatized phosphopeptide enrichment.
• Sequential enrichment and pooling enhance phosphoproteome sensitivity.
• Sequential enrichment by stepwise addition of beads boosts phosphoproteome coverage by 20%.
• Optimized workflow on Orbitrap-Astral yields 32,000 phosphopeptides from 0.5 M cells.

## Graphical Abstract

**Figure:**
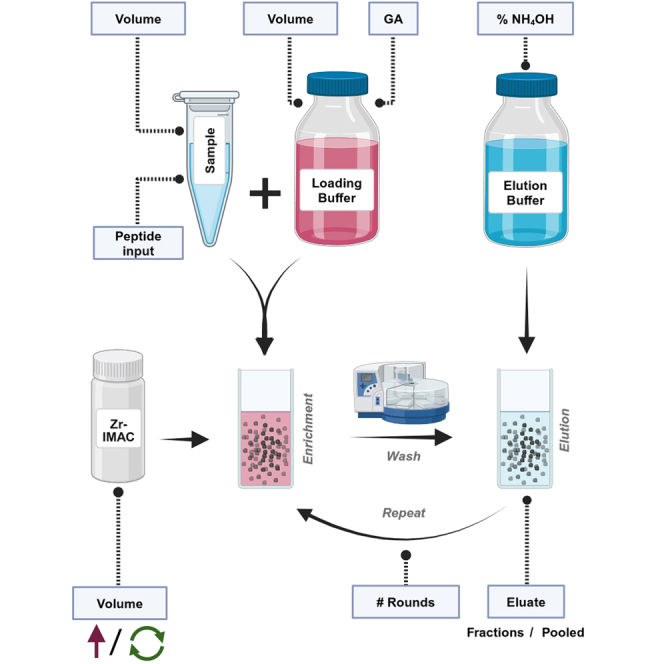

## In Brief

Optimization of experimental parameters for automated on-bead phosphoproteomics sample preparation for low-input samples was conducted. Key factors assessed included glycolic acid concentration, ammonium hydroxide percentage, peptide-to-bead ratio, binding time, and buffer volumes. Using 30 μg of peptides, 16,000 phosphopeptides were confidently identified within 30 min on an Orbitrap Exploris 480, or 32,000 phosphopeptides when using Orbitrap Astral and 0.5 million HeLa cells. An alternative enrichment strategy based on sequential enrichment with stepwise addition of beads boosted coverage by 20%.

Protein phosphorylation is a highly dynamic post-translational modification (PTM) that plays a critical role in regulating cellular signal transduction pathways. Protein phosphorylation has been the objective of extensive studies by the mass spectrometry (MS)-based proteomics community, with the analysis of thousands of phosphorylation sites across different cellular contexts (1, 2, 3, 4, 5). However, there is still ongoing research to try to identify the functionality of most of them (6) since it has been suggested that 75% of the proteome might be phosphorylated (7).⁠ Quantitative mass spectrometry has proved to be the best platform for retrieving large-scale information about the identification, quantification, and localization of phosphorylation sites in complex systems (5). The very deep proteomes described so far⁠ (8) or the recent development of highly sensitive mass spectrometers⁠ (9) indicate the potential to explore the phosphoproteome without the need for specific enrichment of phosphopeptides prior to MS analysis. However, the capacity to explore the functional phosphoproteome is still impacted by the relatively low abundance of phosphorylated peptides and their sub-stoichiometric nature (10). Therefore, to this day, deep phosphoproteomics relies on the enrichment of phosphopeptides prior to LC-MS/MS analysis. In this regard, the phosphoproteomics technology has taken a significant leap in recent years with significant improvements in sensitivity, making it possible to perform phosphoproteomics analysis of minute samples (11), even as low as single spheroids (12). Moreover, the incorporation of magnetic beads into workflows on automated sample preparation platforms nowadays allows for robust high-throughput sample preparation, making phosphoproteomics applicable to large-scale studies (13, 14, 15) and clinical sample cohorts (16). Alternatively, novel high-throughput methods combining suspension trapping on micro-columns with affinity material tips have also allowed for streamlining the sample preparation workflows for phosphoproteomics (17).

Currently, the most popular phosphopeptide enrichment strategies rely on affinity-based methods either by immobilized metal affinity chromatography (IMAC) (18), or metal oxide affinity chromatography (MOAC) (19, 20). Both IMAC and MOAC strategies rely on transition metals (Ti, Zr, Fe) that, either chelated on a substrate (Ti-IMAC or Zr-IMAC), or as metal oxides (TiO2), enable the selective binding of phosphopeptides. The effectiveness of these strategies relies on multiple factors, including the ratio of peptide-to-beads (binding capacity) (21), the loading buffer composition (binding conditions) (22), the washing buffer composition, and sample-bead binding time, among others (23, 24). For instance, the inclusion of competitive non-phosphopeptide binders, such as glycolic acid (GA) or 2,5-dihydoxybenzoic acid (DHB) in the binding buffer, as well as a high concentration of trifluoroacetic acid (TFA), can minimize the binding of highly-acidic or sialic-acid containing peptides (25, 26, 27), significantly increasing the phosphopeptide enrichment efficiency of IMAC and MOAC strategies. Moreover, different strategies based on the binding of phosphopeptides to the metal matrix have been presented to increase the depth and diversity of the purified phosphopeptide population. For instance, Thingholm *et al.* (28) showed that multiply-phosphorylated peptides can be separately purified by sequential elution, based on the higher affinity between the metal matrix and peptides with several phosphate groups. On the other hand, the combination of different bead types has been suggested as a method to enrich complementary sets of phosphopeptides based on their multiplicity and acidity (29).

Moreover, with the advent of data-independent acquisition (DIA) strategies and the implementation of software tools capable of analyzing phosphoproteomics data without the need for spectral libraries (30, 31), the depth obtained from single-shot phosphoproteomics analyses has increased significantly. The combination of short LC gradients with single-shot DIA nowadays provides analysis of deep (phospho)proteomes in a high-throughput manner. In this regard, the improvements achieved by technical sample preparation optimizations have been overshadowed by the increased sensitivity and coverage of detected phosphopeptides by DIA approaches.

In this work, we systematically evaluated how key experimental parameters affect phosphopeptide enrichment with a special focus on low-input samples, which were subsequently analyzed using DIA. We specifically tested the impact of using different (i) bead-to-peptide ratios, (ii) sample-bead-binding times, (iii) concentrations of glycolic acid in the loading buffer, (iv) percentages of ammonium hydroxide (NH_4_OH) in the elution buffer, (v) peptide input amounts, (vi) sample volumes and (vii) loading buffer volumes. To identify the optimal phosphopeptide enrichment conditions, the effects of the different parameters were evaluated in terms of phosphopeptide enrichment efficiency, relative purification of multiply-phosphorylated peptides, and coverage of well-localized phosphosites (class I phosphosites, localization probability >0.75) as a proxy for the quality of the DIA-MS/MS spectra. Then, based on the optimized experimental parameters, we devised different strategies to increase the phosphoproteome depth of the analysis by sequential enrichment strategies, either by repetitive enrichment using the non-bound fraction or by modifying the peptide-to-bead ratio. Finally, we applied our optimized strategy for the phosphoproteomics analysis of a cell dilution series on a state-of-the-art Orbitrap-Astral MS to determine the limits of deep phosphoproteomics analysis with strongly downscaled cell input amounts.

## Experimental Procedures

### Experimental Design and Statistical Rationale

All experiments in this manuscript were performed as experimental replicates. For method optimization on the Orbitrap Exploris, all experiments (from phosphopeptide enrichment to MS analysis) were performed in triplicates (n = 3). For the cell dilution experiment on the Orbitrap Astral, all experiments (from cell lysis to MS analysis) were performed in quadruplicates (n = 4). For the EGF treatment experiment, the experiment was performed using 12 experimental replicates. An overview of all Supplementary Files with experimental data can be found in Table 1.

**Table 1:** Overview of supplemental files

Experiment	Supplemental file
Bead volume	S1
Bead binding time	S2
GA in loading buffer	S3
NH_4_OH in elution buffer	S4
Peptide input amount	S5
Sample volume	S6
Loading buffer volume	S7
Sequential enrichment, new beads	S8
Sequential enrichment, 6 rounds	S9
Sequential enrichment, pooled fractions	S10
Sequential enrichment, increasing beads	S11
HeLa dilution, Astral	S12
HeLa phosphosite annotations	S13

### Cell Culture and Cell Lysis

A549 and HeLa cells were cultured in P15 dishes in DMEM (Gibco, Invitrogen) supplemented with 10% fetal bovine serum (FBS, Gibco) and 100 μg/ml streptomycin (Invitrogen) until 90% confluence was reached.

A549 cells were washed twice with phosphate-buffered saline (PBS) (Gibco, Life Technologies) and lysed using 600 μl 95 °C hot lysis buffer (5% sodium dodecyl sulfate (SDS), 5 mM Tris(2-carboxyethyl)phosphine (TCEP), 10 mM chloroacetamide (CAA), 100 mM Tris pH 8.5). Cells were scraped and collected in a falcon tube and the lysate was incubated at 95 °C, 500 rpm for 10 min.

HeLa cells were first detached with 0.05% trypsin-EDTA (Gibco, Invitrogen) and counted *via* a trypan blue viability assay (10 μl of cell suspension was diluted in 1:1 (v/v) ratio with trypan blue stain 0.4% (v/v)) by using an automated cell counter (Corning). For cell count estimation, the average count of five images acquired with the CytoSMART software was calculated. Cell dilutions with respectively; 1 × 10^6^, 0.5 × 10^6^, 0.2 × 10^6^, 0.1 × 10^6^, 0.05 × 10^6,^ and 0.01 × 10^6^ cells were collected in Eppendorf tubes with four replicates for each condition. Cell pellets were washed with PBS, lysed with 50 μl of 95 °C hot lysis buffer, and incubated at 95 °C, 500 rpm for 10 min.

The lysates were cooled to room temperature and sonicated by microtip probe sonication (Vibra-Cell VCX130, Sonics). Sonication parameters were set to a total runtime of 2 min with pulses of 1 s on and 1 s off at an amplitude of 80%.

For the EGF stimulation experiment, HeLa cells were grown in 24 p6 dishes until 80% confluence. Cells were serum-starved for 6 h upon treatment. Treated HeLa cells were stimulated with 100 ng/ml of EGF for 8 min. Cells were lysed in the plates with 80 μl of boiling lysis buffer (5% SDS, 100 mM Tris pH 8.5, 5 mM TCEP, and 10 mM CAA) and scraped to distribute the lysis buffer among the plates. The lysates were incubated at 95 °C for 10 min with mixing (500 rpm).

### Determination of Protein Concentration *via* BCA-Assay

Protein concentration was determined utilizing the Pierce BCA Protein Assay Kit (Thermo Scientific) according to the manufacturer’s protocol for 96-well plates setup.

For the cell dilution experiment in the Orbitrap Astral data, mean peptide input amounts were estimated based on 25% recovery after PAC digestion from BCA measurement of protein concentration.

### Automatized Protein Aggregation Capture (PAC)-Based Protein Digestion

Protein digestion was performed according to an adapted version of the Protein Aggregation Capture (PAC) based digestion (32) on a KingFisher Flex System (Thermo Scientific) (33) with MagReSyn Hydroxyl beads (ReSyn Biosciences). KingFisher deep-well plates were prepared for washing steps, containing 1 ml of 95% Acetonitrile (ACN) or 70% Ethanol (EtOH). For each sample, 300 μl of digestion solution (50 mM ammonium bicarbonate (ABC)-buffer) containing Lys-C and Trypsin at an enzyme-to-substrate ratio of 1:500 and 1:250, respectively, were prepared and transferred to KingFisher plates. Samples were mixed with 100 mM Tris-buffer to obtain a total volume of 300 μl, transferred to KingFisher plates and ACN was added to a final volume percentage of 70%. The storage solution from the Hydroxyl beads was replaced with 70% ACN. Finally, beads were added to the samples at a protein beads ratio of 1:2. Protein aggregation was carried out in two steps of 1 min mixing at medium speed, followed by a 10 min pause each. Sequential washes were performed in 2.5 min at a slow speed without releasing the beads from the magnet. Digestion was set to 100 cycles of agitation for 45 s and pausing of 6 min overnight at 37 °C. Protease activity was quenched by acidification with TFA to a final volume percentage of 1%.

Processing of HeLa samples was performed similarly but with some adaptations. The ratio of MagReSyn hydroxyl beads to protein was 16:1, the time of digestion was 6 h in 200 μl of 50 mM triethylammonium bicarbonate, and the Lys-C and Trypsin to substrate ratios were 1:100 and 1:50, respectively. After digestion, samples were acidified with 50 μl of 10% formic acid, peptides were concentrated in SpeedVac at 45 °C until 20 μl and directly processed for phosphopeptide enrichment without Sep-Pak desalting.

### Sep-Pak Desalting

Desalting was performed on Sep-Pak C18 cartridges (C18 Classic Cartridge, Waters). The cartridges were conditioned with 900 μl 100% ACN and 3× 900 μl 0.1% TFA followed by sample loading, and washing 3× with 900 μl 0.1% TFA. Peptides were eluted with 150 μl 40% ACN followed by 150 μl 60% ACN. The acetonitrile was evaporated in a SpeedVac at 45 °C and peptide concentration was determined by measuring absorbance at 280 nm on a NanoDrop 2000C spectrophotometer (Thermo Fisher Scientific).

### Phosphopeptide Enrichment

Phosphopeptide enrichment was performed on a KingFisher Flex System (Thermo Scientific) using MagReSyn zirconium-based immobilized metal affinity chromatography (Zr-IMAC HP) beads (ReSyn Biosciences).

#### Standard Phosphopeptide Enrichment Workflow

Samples were mixed with 200 μl loading buffer (80% ACN, 5% TFA, 0.1 M glycolic acid) and transferred to a KingFisher 96 deep-well plate. Additional KingFisher plates were prepared containing 500 μl of loading buffer, 500 μl of washing buffer 2 (80% ACN, 1% TFA), or 500 μl of washing buffer 3 (10% ACN, 0.2% TFA) each. For each sample, 5 μl of beads (20 mg/ml) were suspended in 500 μl 100% ACN previously added to the KingFisher plates. For elution, 200 μl of elution buffer (1% NH_4_OH) was prepared and transferred to KingFisher plates. Beads were washed in loading buffer for 5 min at 1000 rpm, incubated with the samples for 20 min with mixing at medium speed, and subsequently washed in loading buffer, washing buffer 2, and washing buffer 3 for 2 min with mixing at fast speed. Phosphopeptides were eluted from the beads by mixing with elution buffer for 10 min at a fast speed.

When evaluating the effect of different experimental parameters, the standard phosphopeptide enrichment workflow was altered as indicated in the experimental design table (Table 2). For evaluating the influence of varying sample volume while keeping the peptide input the same, samples were diluted with 1% TFA to the final desired concentrations.

**Table 2:** Experimental design for the optimization of phosphopeptide enrichment parameters

Parameter optimization	Peptide input [μg]	Sample V [μl]	LB V [μl]	GA in LB [mol/l]	Beads V [μl]	Bead binding [min]	NH_4_OH in EB [%(v/v)]
Standard	30	3.29	200	0.1	5	20	1
Peptide μg	5–30	Standard	Standard	Standard	Standard	Standard	Standard
Sample V	Standard	7.5–120	Standard	Standard	Standard	Standard	Standard
LB V	Standard	15	100–400	Standard	Standard	Standard	Standard
GA in LB	Standard	Standard	Standard	0–2	Standard	Standard	Standard
Bead V	Standard	Standard	Standard	Standard	1–20	Standard	Standard
Bead binding	Standard	Standard	Standard	Standard	Standard	5–30	Standard
NH_4_OH in EB	Standard	Standard	Standard	Standard	Standard	Standard	0.1–2

Evaluated parameters included peptide input (Peptide μg), sample volume (Sample V), loading buffer volume (LB V), concentration of glycolic acid in the loading buffer (GA in LB), Zr-IMAC HP bead volume (Bead V), beads binding time (Bead binding) and percentage of ammonium hydroxide in the elution buffer (NH_4_OH in EB).

#### Standard Sequential/Looped Enrichment Workflow

Standard sequential enrichment was performed for 2 to 3 enrichment rounds by re-starting the phosphopeptide enrichment workflow on the KingFisher Flex System while keeping samples, beads, and washing buffers the same. For obtaining “pooled” samples from the sequential enrichment, all rounds were carried out re-using the same elution buffer. To retrieve the phosphopeptides captured in each round as separate fractions, the elution buffer was removed after each round and replaced by new elution buffer.

#### Sequential Phosphopeptide Enrichment Workflow Adaptations

Parameters of the sequential phosphopeptide enrichment workflow were altered as indicated in the experimental design table (Table 3). For sequential enrichment with increasing molarity of glycolic acid in the loading buffer, 64 μl loading buffer containing 8 M GA was added to the sample in 200 μl standard (0.1 M GA) loading buffer after the first round of enrichment to adjust to 2 M glycolic acid. Sequential enrichment with additional Zr-IMAC HP beads per round was performed by the addition of 1 μl or 2 μl Zr-IMAC HP beads after the first and second rounds, respectively, to the beads plate containing 1 μl starting amount of beads. Sample-bead incubation times of 1 min and 5 min were applied for testing sequential enrichment in combination with short incubation times. For testing the effect of fresh beads in a sequential approach, Zr-IMAC HP beads were removed after the first round of enrichment, and replaced by the same amount of fresh beads for a second enrichment step.

**Table 3:** Experimental design for the optimization of sequential phosphopeptide enrichment

Sequential	Rounds	Peptide input [μg]	Sample V [μl]	GA in LB [mol/l]	Bead V [μl]	Bead binding [min]	Bead exchange	Collected fractions
Standard	2	30	3.29	0.1	5	20	No	Separate Rounds
6 rounds	6	2.5–20	2.5–20	Standard	Standard	Standard	Standard	Standard
Bead addition	Standard	5–30	Standard	Standard	1 + 1 + 2	Standard	Standard	Pooled & Rounds
New beads	Standard	Standard	Standard	Standard	Standard	Standard	Yes	Standard
Fraction Pooling	Standard	2.5–5	Standard	Standard	Standard	Standard	Standard	Pooled & Rounds

The sequential enrichment workflow design was evaluated in terms of reusing or exchanging the beads after the first enrichment round (New beads), total number of enrichment rounds (6 rounds), addition of beads with increasing enrichment round (Bead addition), and sample pooling approaches for LC-MS/MS analysis (Fractions).

### Sample Preparation for LC-MS/MS Analysis

Eluates containing phosphopeptides were acidified with 40 μl 10% TFA to achieve a pH <2. Acidified eluates were transferred to MultiScreen_HTS_-HV 96-well filtration plates (0.45 μm, clear, non-sterile, Millipore), stacked on 96-well plates, and centrifuged for 1 min at 500*g* to remove in-suspension particles.

Evotip Pure (Evosep) were washed by adding 20 μl 100% ACN and centrifuging for 1 min at 800*g*. Tips were pre-conditioned by the addition of 20 μl 0.1% formic acid (FA) while soaking the tips in 100% isopropanol and centrifuged for 1 min at 800*g*. Filtered samples were added to the tips and loaded by centrifugation for 2 min at 500*g*. Evotip preparation was completed by adding 20 μl of 0.1% FA, centrifuging for 1 min at 800*g*, adding 200 μl of 0.1% FA, and centrifuging for 10 s at 800*g*.

### LC-MS/MS Analysis

Samples were analyzed using an IonOpticks Aurora column (15 cm–75 μm-C_18_ 1.6 μm) interfaced with the Orbitrap Exploris 480 Mass Spectrometer (Thermo Scientific) or the Orbitrap Astral Mass Spectrometer (Thermo Scientific) using a Nanospray Flex Ion Source with an integrated column oven (PRSO-V2, Sonation) to maintain the temperature at 50 °C. In all samples, spray voltage was set to 1.8 kV, funnel RF level at 40, and heated capillary temperature at 275 °C. Samples were separated on an Evosep One LC system using the pre-programmed gradient for 40 samples per day (SPD).

For phosphoproteome analysis of A549 samples using DIA on the Orbitrap Exploris 480 Mass Spectrometer, full MS resolutions were set to 120,000 at m/z 200 and the full MS AGC target was 300% with a maximum injection time (IT) of 45 ms. The AGC target value for fragment spectra was set to 1000%. 49 windows of 13.7 m/z scanning from 472 to 1143 m/z were employed with an overlap of 1 Da. MS2 resolution was set to 15,000, IT to 22 ms, and normalized collision energy (NCE) to 27%.

For phosphoproteome analysis of HeLa samples using DIA on the Orbitrap Astral MS, the MS was operated at a full MS resolution of 180,000 with a full scan range of 480 to 1080 *m/z*. The full scan AGC target was set to 500%. Fragment ion scans were recorded at a fixed resolution of 80,000 and with a maximum IT of 6 ms. 150 windows of 4 m/z scanning from 480 to 1080 m/z were used. The isolated ions were fragmented using HCD with 27% NCE.

### Data Analysis

LC-MS/MS runs were searched using Spectronaut (version 17.1 for Orbitrap Exploris 480 data and version 18 for Orbitrap Astral data) employing a direct DIA search strategy against the Homo sapiens proteome UniProt Database (2022 version, 20,958 entries) supplemented with a database of common contaminants (246 entries). MS2 de-multiplexing was set to automatic. Carbamidomethylation of cysteine was set as a fixed modification, whereas oxidation of methionine, N-terminal protein acetylation, and phosphorylation of serine, threonine, and tyrosine were set as variable modifications. The enzyme/cleavage rule was set to Trypsin/P, the digest type to specific, and a maximum number of two missed cleavages per peptide were allowed. The maximum number of variable modifications per peptide was set to 5 and method evaluation was turned on. PTM localization was turned on, but the localization probability threshold was set to 0. The MS1 and MS2 mass tolerance strategy was set to system default. PSM, peptide, and protein group FDR were set to 0.01 (1%) and the employed directDIA workflow was directDIA+ (Deep). FDR calculation in Specronaut is based on mProphet (34). The precursor q-value and PEP cutoffs were 0.01 and 0.2, respectively. The protein q-value experiment and run wide cutoffs were 0.01 and 0.05, respectively. For quantification, precursor filtering was set to “Identified (Q value)” and performed at the MS2 level using the area. No imputation was performed, and cross-run normalization was turned off. The results were filtered for the best N fragments per peptide between 3 and 6. Decoy generation was performed using the mutated strategy. If not explicitly mentioned, all remaining parameters were set to the default Spectronaut settings.

Searches of data from sequential enrichment sets were performed by searching the enrichment rounds and/or fractions separately while keeping replicates of the same peptide input amount in the same analysis.

Precursor-level pivot tables were exported from Spectronaut for phosphopeptide analysis. Tables were filtered to contain unique modified sequences (*i.e.*, phosphopeptide isomers—same stripped sequence but different phosphorylation site—are kept as separated entities) and unique modified sequences containing phosphorylation sites were further filtered to preserve only those with a localization score >0.75 in at least one replicate. The unfiltered precursor pivot tables are available as Supplemental Files, which have been deposited to the ProteomeXchange Consortium *via* the PRIDE partner repository with the dataset identifier PXD045601. An overview of all Supplementary Files can be found in Table 1.

Data was exported in long-format and imported into Perseus (v1.6.5.0) where it was collapsed into phospho-sites or phosphopeptides using the “peptide-collapse” plugin (v1.4.2) described in Bekker-Jensen *et al.* (35). For collapse into phosphosites, the option “Target PTM site-level” was used. By default, the localization cutoff was kept at 0.75. When evaluating the localization probability distribution, the localization cutoff was set to 0. Importantly, phopshosites reported by “peptide-collapse” plugin must have been identified and/or localized in at least two experimental replicates. Collapse into phosphopeptides using the Perseus plugin was employed for quantification purposes. For phosphopeptide collapse, the option “ModSpec peptide-level” was used and localization cutoff was kept at 0.75. Phosphopeptide collapse in Perseus groups together different phospho-isomers.

All remaining processing steps were performed either in Perseus (v1.6.5.0) or R (v3.6.2 or higher) implementing the packages ComplexHeatmap (36), sitools (https://CRAN.R-project.org/package=sitools), eulerr (https://CRAN.R-project.org/package=eulerr), stringr (https://CRAN.R-project.org/package=stringr), ggplot2 (37), dplyr (https://CRAN.R-project.org/package=dplyr) and tidyverse (38). Calculation of isoelectric point (pI) values was performed using the package pIR (http://github.com/ypriverol/pIR2015), considering N-terminal acetylation and phosphorylation of the peptides.

Network analysis was performed in Cytoscape (39) (v3.10.1) using SIGNOR App (40) (v1.2) to import the EGFR signaling pathway and Omics Visualizer (41) (v1.3.1) to add the phosphosite information to each node of the network. Regulatory sites of transcription factors in the dataset were determined based on the “PG ProteinDescriptions” column of the Spectronaut report and the regulatory site annotation obtained from Perseus using PhosphoSitePlus database.

## Results and Discussion

### Phosphopeptide Binding Conditions Affect the Population of Purified phosphopeptides

**Fig. 1:**
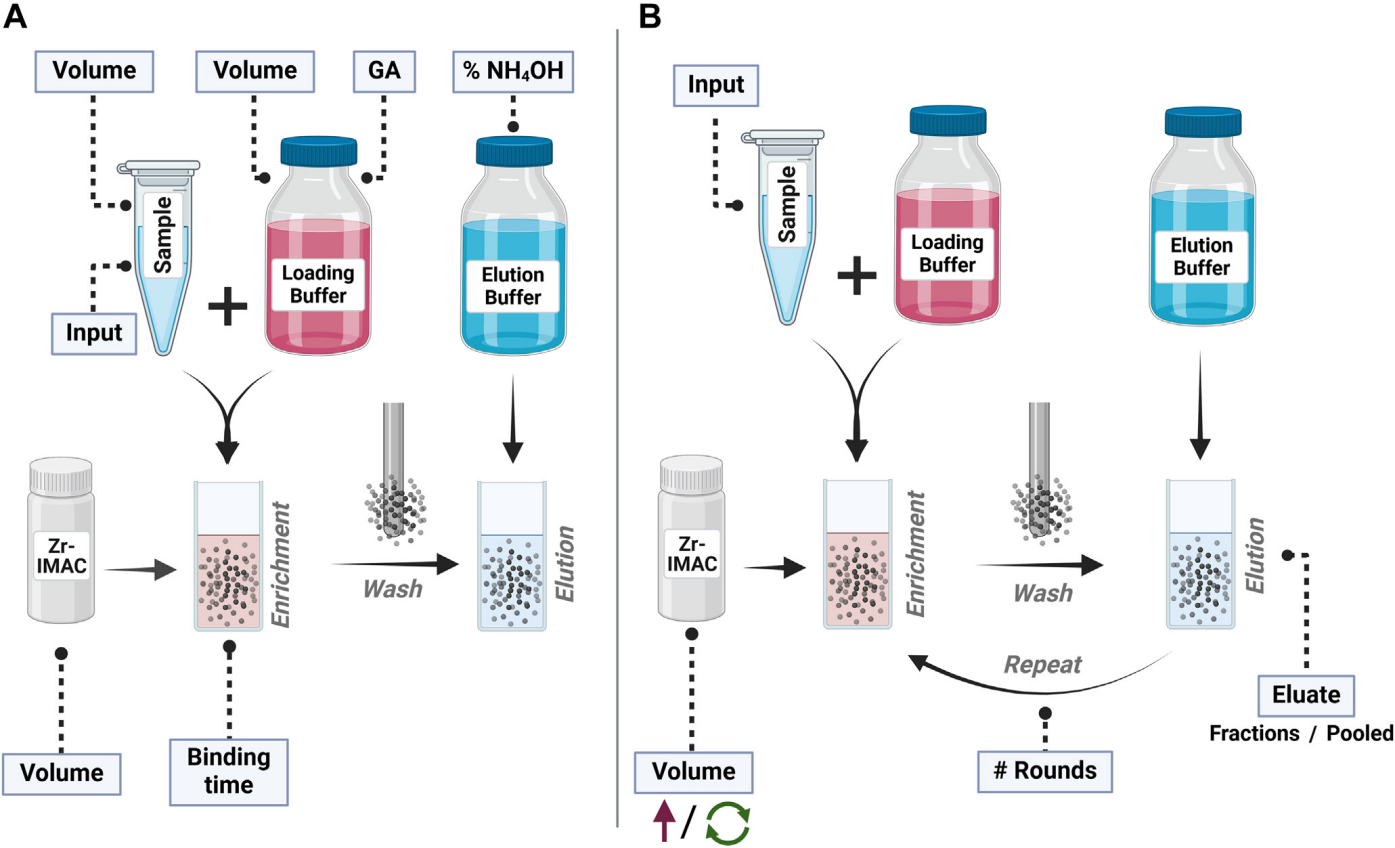
**Experimental design.***A*, schematic overview of the phosphopeptide enrichment workflow and the evaluated experimental parameters. Evaluated parameters included the peptide input, the sample volume, the loading buffer volume, the proportion of glycolic acid in the loading buffer, the percentage of ammonium hydroxide in the elution buffer, the Zr-IMAC HP bead volume, and the sample-beads binding time. *B*, schematic overview of the sequential phosphopeptide enrichment workflow and the evaluated experimental parameters. Evaluated parameters included the peptide input amount of the sample, the number of sequential enrichment rounds and the way of retrieving the eluate by either reusing the elution buffer and obtaining a “Pooled” eluate or exchanging the elution buffer after each round and obtaining each enrichment round as single fraction for LC-MS/MS analysis. Modified sequential enrichment approaches included exchanging, reusing or increasing the Zr-IMAC HP bead volume.

To establish an optimized automated phosphopeptide enrichment procedure for high-sensitivity samples (13), we started by evaluating the main experimental parameters that can affect phosphopeptide enrichment. This was done by introducing modifications to our default automated protocol for sensitive phosphoproteomics, which relies on the use of magnetic Zr-IMAC HP beads in the KingFisher System. The resulting phosphopeptide mixtures were subsequently analyzed by DIA-MS on an Orbitrap Exploris 480 MS coupled to an Evosep One LC system taking advantage of the higher sensitivity of the Whisper gradients. All enrichments were performed from a starting amount of 30 μg of purified peptides from a whole cell tryptic digest of the A549 lung cancer cell line, as it represents the optimal peptide input amount for phosphopeptide enrichment and subsequent analysis with our LC-MS/MS setup using Whisper gradients. By using this amount, we ensured to have an adequate reference to assess the effect of the changes in the experimental workflow. The phosphopeptide enrichment protocol consists of three main steps: (i) the binding of phosphopeptides to the beads, (ii) the washing of the beads to remove non-specific interactions, and (iii) the elution of phosphopeptides from the beads (Fig. 1*A*). We focused on the first part of the protocol, the binding of phosphopeptides to the beads, in which we evaluated the following parameters: (i) the beads to peptide ratio, (ii) the proportion of glycolic acid in the loading buffer, (iii) the binding time, and (iv) the sample to loading buffer volume ratio. In the last step of the protocol, we evaluated the effect of modifying the concentration of ammonium hydroxide in the elution buffer (Fig. 1*B* and Table 2).

**Fig. 2:**
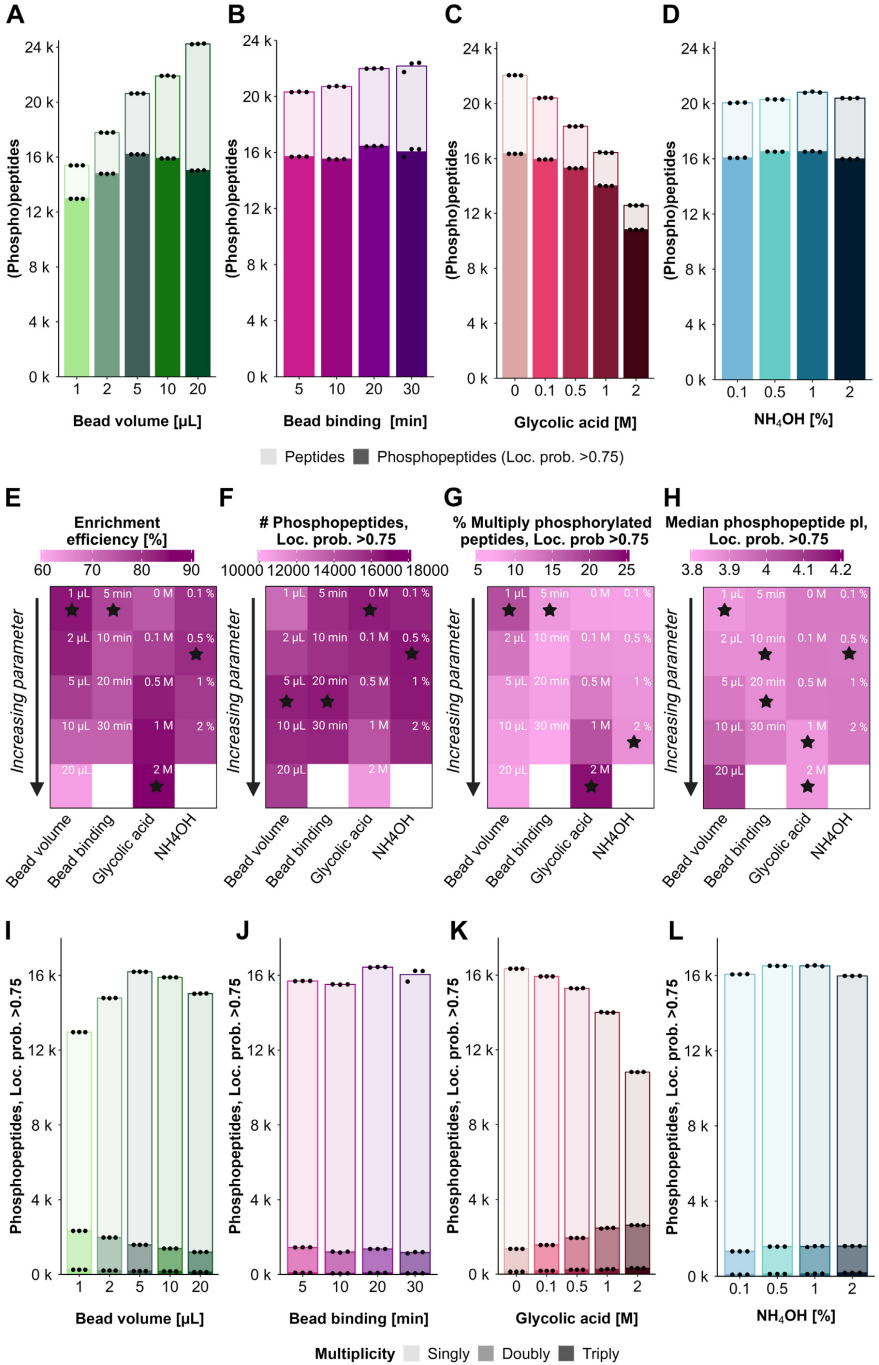
**Evaluation of experimental parameters during phosphopeptide enrichment.***A*–*D*, barplots show the mean numbers of peptides (*light color*) and phosphopeptides with loc. prob. >0.75 (*dark color*) identified across three experimental replicates using different (*A*) molarities of glycolic in the loading buffer, (*B*) percentage of ammonium hydroxide in the elution buffer, (*C*) Zr-IMAC HP bead volumes or (*D*) sample-bead binding times. Each dot represents one experimental replicate. *E*–*H*, Heatmaps show the influence of increasing molarity of glycolic acid in the loading buffer, percentage of ammonium hydroxide in the elution buffer, Zr-IMAC HP bead volume or sample-bead incubation time on (*E*) selectivity of the enrichment in terms of identified phosphorylated and non-phosphorylated peptides (*F*) number of identified phosphopeptides with loc. prob. >0.75 (*G*) percentage of multiply-phosphorylated peptides with loc. prob. >0.75 in the context of total identified phosphorylated peptides with loc. prob. >0.75 (*H*) median pI of phosphopeptides with loc. prob. >0.75. Stars refer to the highest value within the respective parameter column for (*E*–*G*) and to the lowest value for (*H*). If not otherwise indicated, all values represent the mean of three experimental replicates. *I*–*L*, barplots show the mean numbers of singly (*light color*), doubly (*medium color*), and triply (*dark color*) phosphorylated peptides with loc. prob. >0.75 identified across three experimental replicates using different (*I*) molarities of glycolic in the loading buffer, (*J*) percentage of ammonium hydroxide in the elution buffer, (*K*) Zr-IMAC HP bead volumes, or (*L*) sample-bead binding times. Each dot indicates one experimental replicate.

The proteomics community has extensively evaluated the beads-to-peptide ratio as this plays an important role in the phosphopeptide enrichment efficiency. A high beads-to-peptide ratio can lead to increased binding of non-phosphorylated peptides due to nonspecific interactions with the bead surface, thus reducing the selectivity of the enrichment. In contrast, it has also been described that a too low beads-to-peptide ratio can result in a higher fraction of multiply-phosphorylated peptides identified (21). In this work, we assessed the effect of using different bead amounts. This is particularly relevant when performing enrichment with low peptide amounts since it is difficult to proportionally scale down the volume of beads, due to lack of reproducibility when pipetting low volumes of beads. Therefore, starting from 30 μg of peptides, we evaluated what the best compromise between bead volume and good phosphopeptide recovery would be by testing 1, 2, 5, 10, and 20 μl of beads corresponding to a beads-to-peptide ratio of 0.7, 1.3, 3.3, 6.7 and 13.3 (Fig. 2*A*, supplemental Table S1, and supplemental File S1). We observed that the best outcome was obtained using a volume of 5 μl (beads-to-peptide ratio of 3.3), which resulted in 16,193 phosphopeptides. Importantly, throughout this work, we only report a phosphopeptide as valid for those where the phosphorylation was localized to an amino acid with a score >0.75. When investigating enrichment efficiency, it should be noted that there are different ways of calculation: either based on counts (*i.e.*, number of phosphopeptides *versus* total number of peptides), or based on abundance (*i.e.*, MS signal from phosphopeptides *versus* total signal). In most figures in this work (unless explicitly stated), enrichment efficiencies are reported based on counts. However, this strategy can result in a biased interpretation of the quality of the phosphopeptide enrichment performed. For instance, when comparing enrichment efficiency based on counts with enrichment efficiency based on abundance in the samples of our bead volume evaluation, we found that the enrichment efficiency based on abundance was consistently higher than that based on counts for all tested bead volumes (supplemental Fig. S1), and in all cases >90%, reflecting that the enrichment strategy prioritized phosphopeptides (supplemental Fig. S2) as expected and on par with previous works (13, 17, 22). We suggest that this divergence could be a consequence of using a DIA search strategy, which can favor the identification of non-phosphopeptides, even at low abundance levels. However, we used count-based enrichment since it gives more insights into the nuances between the parameters tested. For instance, increasing the bead volume to 20 μl slightly decreased the overall number of phosphopeptides to 15,026 as well as the relative enrichment efficiency based on counts (from 79% with 5 μl to 62% with 20 μl), whilst at abundance level, the enrichment efficiency changed from 98 to 93%. Moreover, reducing the bead volume to 1 μl resulted in higher enrichment efficiency (84%) (Fig. 2*E*) and a trend towards more acidic phosphopeptides (Fig. 2*H*), although lower overall phosphoproteome coverage (12,962 phosphopeptides) (Fig. 2, *A* and *F*). Our titration experiment also confirmed that the selectivity of the enrichment in regard to the purification of mono- or multiply-phosphorylated peptides is affected by the available binding surface, as the absolute number of multiply-phosphorylated peptides increased with decreasing bead amount (Fig. 2, *G* and *I*).

We next evaluated the effect of changing the beads-to-peptide binding time to explore whether shortening the binding time would lead to lower phosphoproteome depth and/or bias the recovery towards multiply-phosphorylated peptides. Shortening the binding time from 20 to 5 min had a slight impact on the phosphoproteome depth achieved, with 15,700 phosphopeptides quantified after 5 min binding compared to 16,440 after 20 min binding (Fig. 2, *B* and *F*, supplemental Table S2, and supplemental File S2). However, it seems that there was a slight improvement in enrichment efficiency (from 75% in 20 min to 77% in 5 min), likely due to more unspecific binding to the beads with longer incubation time (Fig. 2*E*). Moreover, only a marginal increase in the percentage of multiply-phosphorylated peptides was observed by shortening the binding time (Fig. 2, *G* and *J*).

The use of non-phosphopeptide excluders during binding to prevent binding of non-phosphopeptides with high affinity towards IMAC-metal conjugates was evaluated next. In our standard protocol, we originally used 0.1 M of GA as a competitive binder in the loading buffer, as recommended by the beads’ manufacturer. With this GA concentration, we obtained an enrichment efficiency of 78% based on peptide counts or 94% based on MS signal abundance. In line with others (22), we observed that increasing the GA concentration up to 2 M improved the overall enrichment efficiency (86% based on peptide counts) and slightly lowered the median phosphopeptide pI (Fig. 2*H*), but at the cost of reduced phosphoproteome depth with 10,811 phosphopeptides quantified against 15,929 in our standard protocol (Fig. 2, *C* and *F*, supplemental Table S3, and supplemental File S3). In contrast, removing the GA greatly decreased the enrichment efficiency (to 74% based on peptide counts), while preserving the phosphoproteome coverage (16,342 phosphopeptides with 0 M GA) obtained with 0.1 M of GA (Fig. 2, *C*–*F*). Interestingly, the high concentrations of GA (1 M and 2 M) biased the enrichment towards multiply-phosphorylated peptide species, providing up to 16% more multiply-phosphorylated peptides than 0.1 M of GA (Fig. 2, *G* and *K*). This demonstrated that in high concentrations, GA does not only compete with acidic amino acids in its function as a competitive binder, but also with phosphopeptides. Hence, the most competitive phosphopeptides (multiply phosphorylated species) would bind preferentially. Overall, we could confirm that multiply-phosphorylated peptides have a higher affinity towards Zr-IMAC HP beads, explaining why they are preferably recovered with more competitive binding conditions such as 2 M GA or shorter binding time.

Next, we questioned whether the lower multiply-phosphorylated peptide recovery in standard enrichment conditions (0.1 M GA, 20 min binding time) could be due to the stronger binding of multiply-phosphorylated peptides to the beads, preventing them from proper elution when present in a large pool of singly-phosphorylated peptides. We evaluated different concentrations of ammonium hydroxide (NH_4_OH) for phosphopeptide elution from the beads, ranging from 0.1 to 2% (v/v) (supplemental Table S4 and supplemental File S4). Although subtle, we observed that the lowest NH_4_OH concentration tested (0.1%) resulted in lower multiply-phosphorylated peptide recovery with 8% multiply-phosphorylated phosphorylated peptides compared to 10% multiply-phosphorylated peptides with 2% NH_4_OH (Fig. 2, *D* and *L*). Enrichment efficiency was highest with 0.5% NH_4_OH (81%) and slightly decreased with higher NH_4_OH concentrations (79% with 1% NH_4_OH and 78% with 2% NH_4_OH) (Fig. 2, *D*–*E*). Altogether, we conclude that the percentage of NH_4_OH in the elution buffer does not significantly impact the elution of bound phosphopeptides from the beads.

**Fig. 3:**
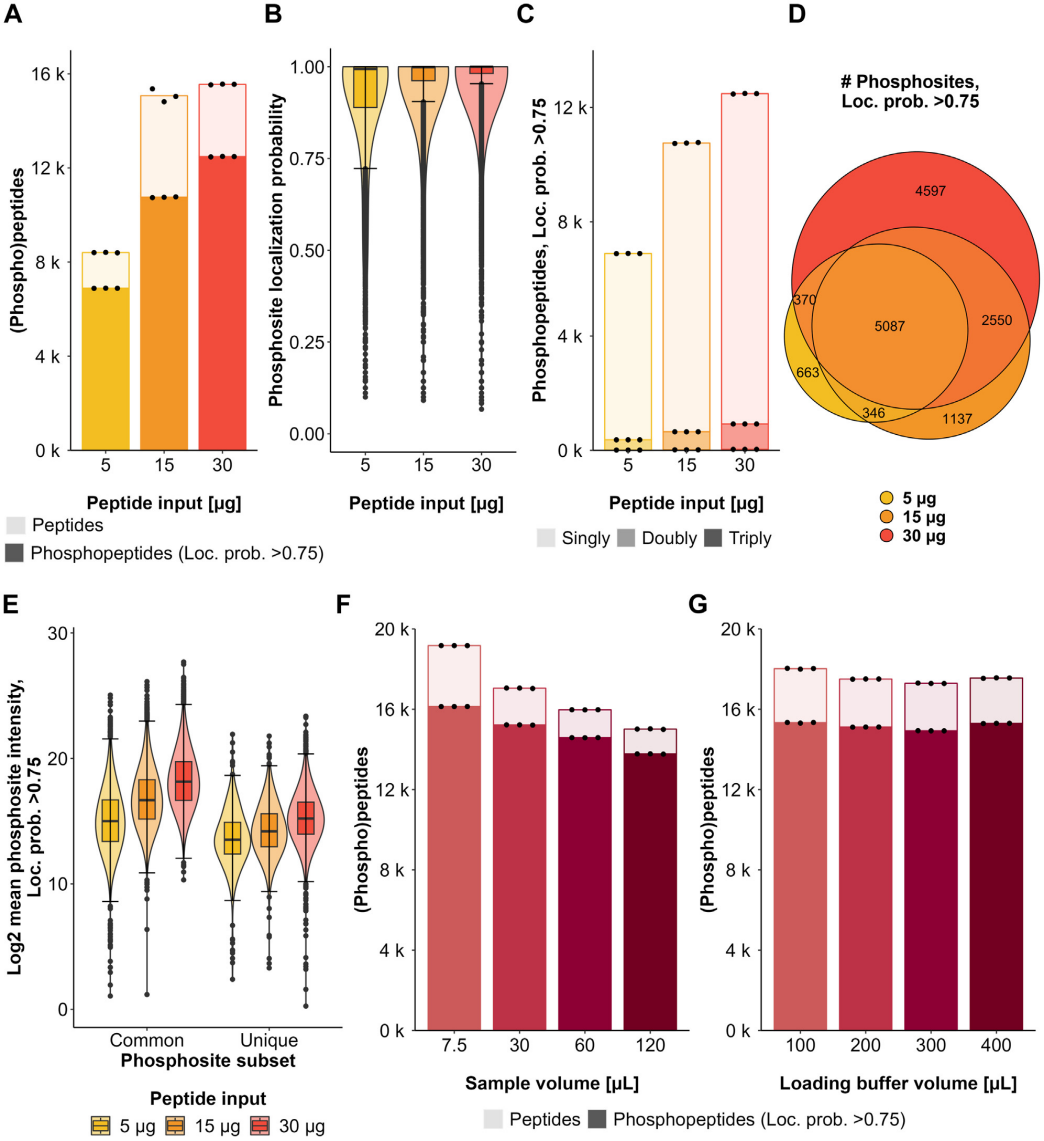
**Evaluation of peptide input and sample/loading buffer volume effects.***A*, barplots show the mean number of peptides (*light color*) and phosphopeptides with loc. prob. >0.75 (*dark color*) identified across three experimental replicates using different peptide input amounts. Each dot represents one experimental replicate. *B*, violin plots show the range and distribution of the localization probability of phosphosites identified using different peptide input amounts. *C*, barplots show the mean numbers of singly (*light color*), doubly (*medium color*), and triply (*dark color*) phosphorylated peptides with loc. prob. >0.75 was identified across three experimental replicates using different peptide input amounts. Each dot represents one experimental replicate. *D*, the Venn diagram shows uniquely and commonly identified phosphosites with loc. prob. >0.75 among different peptide input amounts. *E*, Violin plots show the log2 mean intensities of uniquely and commonly identified phosphosites with loc. prob. >0.75 among different peptide input amounts. *F*, barplots show the mean numbers of peptides (*light color*) and phosphopeptides with loc. prob. >0.75 (*dark color*) identified across three experimental replicates using the same peptide input amount (30 μg) diluted in different sample volumes, mixed with the same volume of loading buffer (200 μl). Each dot represents one experimental replicate. *G*, barplots show the mean numbers of peptides (*light color*) and phosphopeptides with loc. prob. >0.75 (*dark color*) identified across three experimental replicates using the same peptide input amount (30 μg) diluted in the same sample volume (15 μl), mixed with different volumes of loading buffer. Each dot represents one experimental replicate.

To test the influence of the sample input amount in the low peptide range, we performed phosphopeptide enrichment using 30, 10, and 5 μg of peptides. As expected, phosphopeptide recovery is highly dependent on the sample input with 6888 phosphopeptides quantified from 5 μg compared to 12,480 phosphopeptides quantified using 30 μg of peptide amount (Fig. 3*A*, supplemental Table S5, and supplemental File S5). Also, the site localization scores scaled with sample amount (Fig. 3*B*), reflecting that the capacity of the search engine to localize phosphorylation sites is dependent on the signal and therefore the quality of the MS2 spectra. The percentage of multiply-phosphorylated peptides barely increased with increasing peptide amount (5% for 5 μg, 6% for 15 μg and 7% for 30 μg) (Fig. 3*C*). Finally, phosphopeptides enriched from different peptide input amounts highly overlapped, with more abundant phosphopeptides being preferentially enriched from all input amounts, and the phosphoproteome depth increased as expected with higher input amounts, where lower abundant phosphopeptides were detectable (Fig. 3, *D* and *E*).

Finally, we evaluated the sample volume-to-loading buffer (LB) volume ratio. First, we diluted the sample while keeping the LB volume constant (Table 2, Fig. 3*F*, supplemental Table S6, and supplemental File S6), and second, we kept the sample highly concentrated in a constant volume of 15 μl while using increasing LB volumes (Table 2, Fig. 3*G*, supplemental Table S7, and supplemental File S7). Increasing the ratio sample volume-to-LB volume had a negative impact on the phosphoproteome depth (16,132 phosphopeptides with 7.5 μl sample volume *versus* 13,770 phosphopeptides with 120 μl sample volume), potentially due to the dilution of the LB by addition of higher sample volumes during binding. However, the dilution of LB in increasing volumes of the sample had an impact on the enrichment by reducing the binding of non-phosphorylated species (2691 non-phosphorylated peptides with 7.5 μl sample volume *versus* 2258 non-phosphorylated peptides with 120 μl sample volume) (Fig. 3*F*). On the other hand, we observed that the volume of the loading buffer did not have an impact on the phosphoproteome depth or enrichment efficiency, as long as the sample was kept to a minimal volume (<30 μl) (Fig. 3*G*).

Altogether, in Zr-IMAC HP-based phosphopeptide enrichment, we observed that the beads-to-peptide ratio, the concentration of GA in the loading buffer, the peptide input itself, as well as the sample concentration can have a significant impact on the resulting phosphoproteomes. On the contrary, the binding time and percentage of NH_4_OH in the elution buffer did not seem to have such a significant influence on the phosphopeptide enrichment.

Our evaluation showed that for highly sensitive phosphoproteomics, 5 μl of Zr-IMAC HP beads, 20 min binding time, 0.1 M GA in the loading buffer and 0.5% of NH_4_OH in the elution buffer should be employed to obtain the best phosphopeptide enrichment. However, when multiply phosphorylated peptides are of interest, highly competitive binding conditions such as using 1 μl of Zr-IMAC HP beads, 5 min binding time, 2 M GA in the loading buffer and a high percentage of NH_4_OH in the elution buffer (2%) could be the best choice.

### Sequential Enrichment of the Phosphoproteome as a Strategy to Increase the Depth of the Analysis

Our data so far showed that changing experimental parameters during phosphopeptide enrichment can have a significant impact on the population of enriched phosphopeptides. Exploring these differences has been suggested before as a potential way to enhance the performance of phosphopeptide enrichment strategies by performing sequential enrichment. The most straightforward way to perform sequential enrichment is to iterate the enrichment by using the flow-through from the previous enrichment. This strategy has been utilized before to increase the depth of the phosphoproteome (14, 24, 42, 43). Therefore, we wanted to evaluate how many sequential rounds of enrichment are needed to efficiently deplete a sample for phosphopeptides, and whether sequential enrichment is as efficient with high peptide input amounts as it is with low peptide input amounts.

First, we evaluated whether the beads employed in one round of phosphopeptide enrichment could be reused for a second enrichment. Our data reflected the potential of reusing the beads for sequential enrichment. Interestingly, reusing the beads from the first enrichment in a second one resulted in a higher phosphopeptide recovery in the second enrichment round (10,928 phosphopeptides with new beads compared to 12,812 phosphopeptides with reused beads) and a better enrichment efficiency, when compared to using new beads for the second enrichment (52% with new beads compared to 60% with reused beads) (supplemental Fig. S3, supplemental Table S8, and supplemental File S8).

**Fig. 4:**
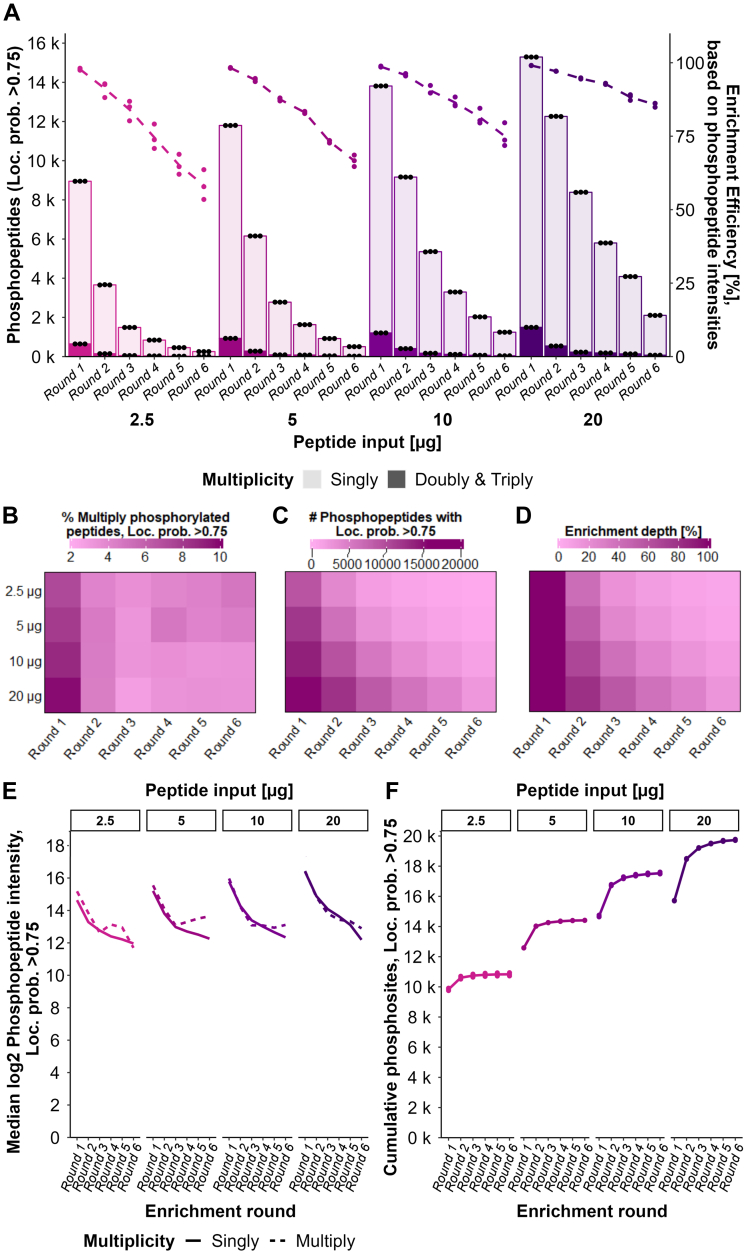
**Evaluation of an extensive 6-round sequential enrichment approach.***A*, barplots show the mean numbers of singly phosphorylated (*light color*) and multiply-phosphorylated (*dark color*) peptides with loc. prob. >0.75 (*dark color*) identified across three experimental replicates using different peptide input amounts for a sequential six-round enrichment. Each fraction (round) was obtained as eluate after the respective enrichment round and analyzed separately *via* LC-MS/MS. Each dot represents one experimental replicate. *Line plots* represent the mean enrichment efficiency across three experimental replicates in each round per peptide input amount based on phosphopeptide intensities in percentage. Each dot represents one experimental replicate. *B*–*D*, Heatmaps show, for each peptide input amount and enrichment round, the (*B*) percentage of multiply-phosphorylated peptides with loc. prob. >0.75 in the context of the total number of identified phosphorylated peptides with loc. prob. >0.75 (*C*) number of identified phosphopeptides with loc. prob. >0.75 in the context of total identified phosphorylated peptides (*D*) enrichment depth in percentage in terms of number of identified phosphopeptides with loc. prob. >0.75 relative to round 1 of the respective peptide input amount. *E*, *line plots* show the medians of log2 mean intensities across replicates of singly and multiply-phosphorylated peptides with loc. prob. >0.75 identified in each enrichment round upon different peptide input amounts. *F*, *line plots* represent the mean number of cumulative phosphosites with loc. prob. >0.75 per peptide input amount and enrichment round across three experimental replicates. “Cumulative” refers to the addition of phosphosites that were not identified in the previous enrichment round(s). Each dot represents one experimental replicate.

Next, we tested more extensive sequential enrichment (up to six rounds) for samples spanning 20 to 2.5 μg of peptide input. Whilst the enrichment efficiency (based on phosphopeptide intensities, measured as the percentage of the overall identified MS signal intensity from phosphopeptides alone) was above 90% in the first enrichment round for all amounts tested, it abruptly decreased with lower peptide input amounts in subsequent enrichment rounds down to 86% for 20 μg and 58% for 2.5 μg in round six (Fig. 4*A*, supplemental Fig. S4, *A* and *B*, and supplemental File S9). Similarly, the phosphoproteome depth (phosphopeptide count relative to round 1) decreased with each sequential enrichment, which was more evident for lower input amounts (Fig. 4, *A*, *C*, and *D*, and supplemental Table S9). The number of additional unique phosphopeptides did not significantly increase after the third enrichment round (Fig. 4*F* and supplemental Fig. S4*A*), even though up to 2105 phosphopeptides were still enriched in the sixth enrichment of 20 μg peptide input amount (Fig. 4, *A*–*C*). The population of phosphopeptides enriched in each subsequent enrichment, especially from third and forward, was mainly driven by abundance since the most abundant peptides in the first enrichment round continued being enriched subsequently (supplemental Fig. S4*A*). Unlike the phosphopeptides, the non-phosphorylated peptides eluted differently across the sequential enrichments (supplemental Fig. S4*A*). At least three different trends were observed in the non-phosphorylated peptides elution: (group 1) peptides eluting mainly in the first fraction (enrichment round), (group 2) peptides eluting mainly in the second fraction, and (group 3) peptides with a consistent elution between fractions (supplemental Fig. S4*A*). We decided to explore the nature of these peptides further to understand the mechanisms behind the non-phosphopeptide binding with Zr-IMAC HP beads. When compared to a comprehensive proteome of the same cell line (A549), it was evident that the non-phosphorylated peptides that bound to the beads belonged to the most abundant pool of peptides in the proteome (supplemental Fig. S4*C*). In particular, the non-phosphorylated peptides that showed a consistent elution across the first three fractions (group 3) were more abundant in the proteome than the rest, showing that their binding is most likely mainly driven by abundance (supplemental Fig. S4*C*). We also calculated the isoelectric point of these peptides and found them to be generally acidic (pI ∼5), although less than the phosphopeptides (pI ∼3) (supplemental Fig. S4*D*). Altogether, these data show that although the selectivity will strongly favor the binding of phosphopeptides, the high abundance of non-phosphorylated peptides can lead to unspecific binding during sequential enrichment.

Throughout the course of this project, we observed that the way DIA proteomics data, and in our case phosphoproteomics data in particular, is analyzed by the search engine (*i.e.*, Spectronaut) had an impact on the identifications. In spectral library-free mode (direct-DIA) searches in Spectronaut, when several files are searched together, the information from all of them is used during the search, allowing data for spectral library inference to be obtained from one file and used during peptide identification in the other files. This effect is of special relevance when searching together high-load samples and low-load samples, and it is a strategy often employed in the field of single-cell proteomics to boost identifications. In our experiments, searching all six fractions together using direct-DIA led to an increase in the number of identified (phospho)peptides in all fractions (supplemental Fig. S4*E*). In contrast, when the search of each enriched fraction was done separately using the evaluation mode, most of the identified peptides were found uniquely in the first enrichment (supplemental Fig. S4*F*).

We previously observed that the site localization score decreased with lower peptide input and hence phosphopeptide intensity (Fig. 3*B*). Since we observed a constant decrease in median phosphopeptide intensity with each subsequent enrichment (Fig. 4*E*), we evaluated whether the site localization scores also worsened in each subsequent enrichment (supplemental Fig. S5, *A*–*D*). Interestingly, such a trend was not observed for lower input amounts (supplemental Fig. S5, *C* and *D*). Potentially, this could be due to the lower number of phosphopeptides identified in the last enrichment rounds when starting with 2.5 or 5 μg, which likely represents the more abundant phosphopeptides which are therefore easier to localize (supplemental Fig. S6*A*). Conversely, for higher input amounts, the population of phosphopeptides in the last enrichment might include less abundant phosphopeptides that result in worse localization scores.

Finally, we hypothesized that sequential enrichment might eventually deplete the most abundant phosphopeptides, allowing other phosphopeptide species to be enriched. Interestingly, we observed that while the overall intensity in the population of singly phosphorylated peptides decreased over time, the multiply-phosphorylated counterpart increased towards the last fractions, especially for low input amounts (Fig. 4*E* and supplemental Fig. S6). Although the number of multiply-phosphorylated peptides decreased with each enrichment (Fig. 4*B*), this increment in the intensity of multiply-phosphorylated peptides could be due to the higher affinity of those peptides when the overall population of phosphopeptides is depleted.

**Fig. 5:**
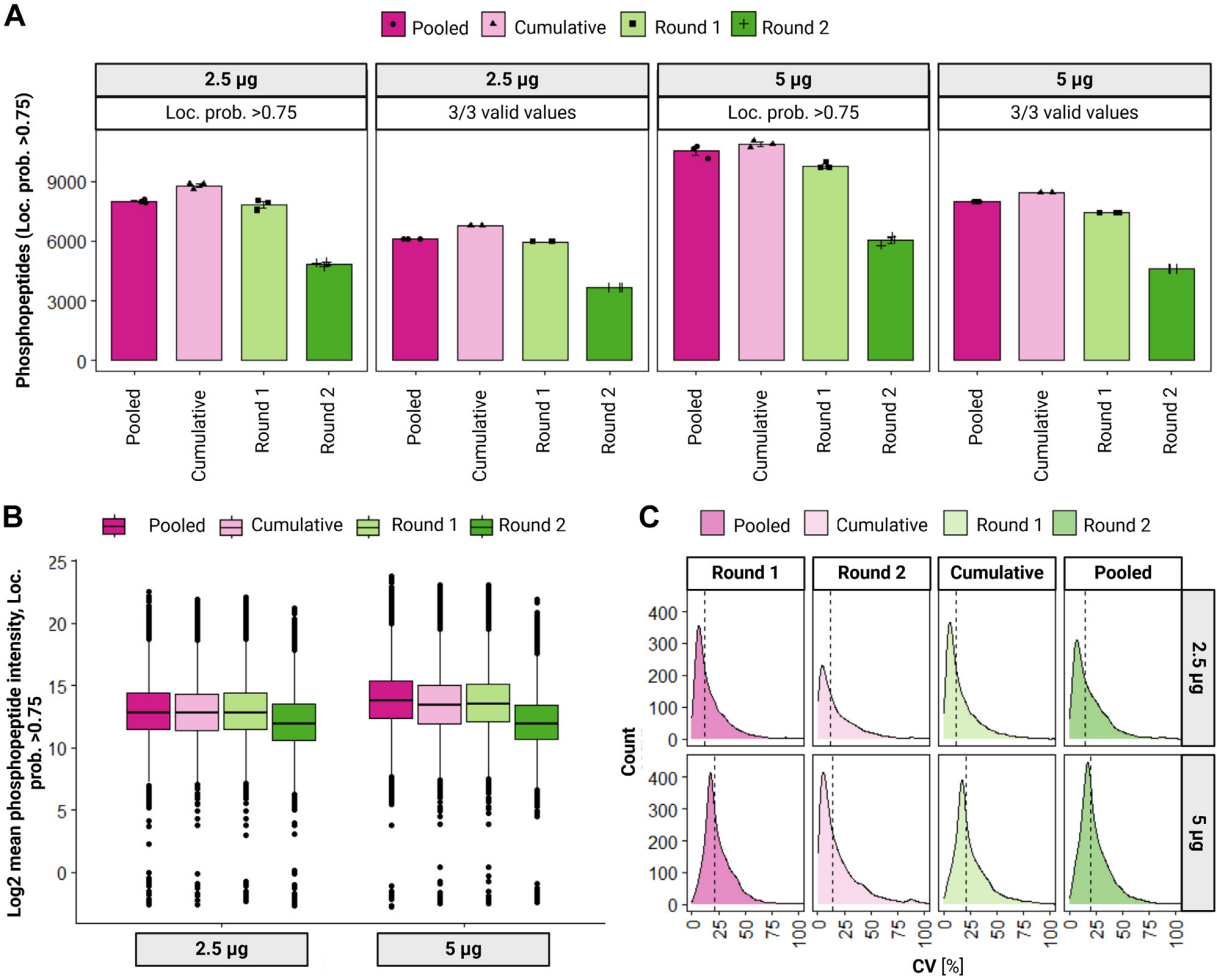
**Strategies for pooling sequential enrichment samples**. *A*, barplots show the numbers of phosphopeptides with loc. prob. >0.75 (dark color), 3/3 valid intensity values among replicates (medium color) or with a CV <0.2 among replicate intensities (*light color*) identified in a two-round sequential enrichment approach using 2.5 μg (pink) or 5 μg (*purple*) peptide input amount. Each dot represents one replicate. Each fraction (round) was either obtained as eluate after the respective enrichment round and analyzed separately *via* LC-MS/MS (“Round 1” and “Round 2”) or obtained as a pooled eluate by reusing the elution buffer from the first enrichment round (“Pooled”). “Cumulative” refers to the cumulation of unique phosphopeptide IDs identified in the separate fractions (“Round 1” & “Round 2”) during data analysis. *B*, boxplots show the log2 mean intensities of identified phosphopeptides with loc. prob. >0.75 per fraction and peptide input amount. *C*, density plots show the distribution of CVs across replicates of phosphopeptide (loc. prob. >0.75) intensities per fraction (columns) and peptide input (rows) after normalization. The labels within the density plots show the median CVs.

Overall, the highest increase in phosphoproteome depth when doing sequential enrichment originated mostly from the second enrichment (Fig. 4, *D* and *F*). However, doing sequential enrichment involves not only more sample preparation time, but also an increase in subsequent MS measurement time. Moreover, there is no standardized approach on how to handle multiple enrichments from a quantitative phosphoproteomics perspective, considering the high redundancy of phosphopeptides identified across the sequentially enriched fractions and that their intensity is relative to their environment. Therefore, we hypothesized that a potential solution to benefit from the increase in depth of sequential enrichment could be achieved by pooling the fractions prior to MS analysis. We explored this possibility for highly sensitive analysis, using 2.5 and 5 μg of peptide as starting amounts for enrichment. Interestingly, we observed a gain of 7% (from 9750 phosphopeptides in one single enrichment to 10,511 phosphopeptides in a pooled sample, supplemental Table S10, supplemental File S10) when combining first and second enrichment from 5 μg of input peptides (Fig. 5*A*). The gain in IDs in the pooled sample could be potentially due to a boost in the intensity (Fig. 5*B*). However, we did not observe such a significant gain when using 2.5 μg of peptide (only 2% increase in phosphopeptides) (Fig. 5, *A* and *B*). Furthermore, we explored the impact of pooling from a quantitative perspective by calculating the CVs of the pooled fractions and comparing it to separate enrichments or the cumulative strategy (*i.e.*, First and second enrichment analyzed separately by LC-MS/MS, and the resulting data merged in-silico afterward). Reassuringly, CVs were not significantly affected by pooling the samples and remained with a median of around 20% (Fig. 5*C*).

**Fig. 6:**
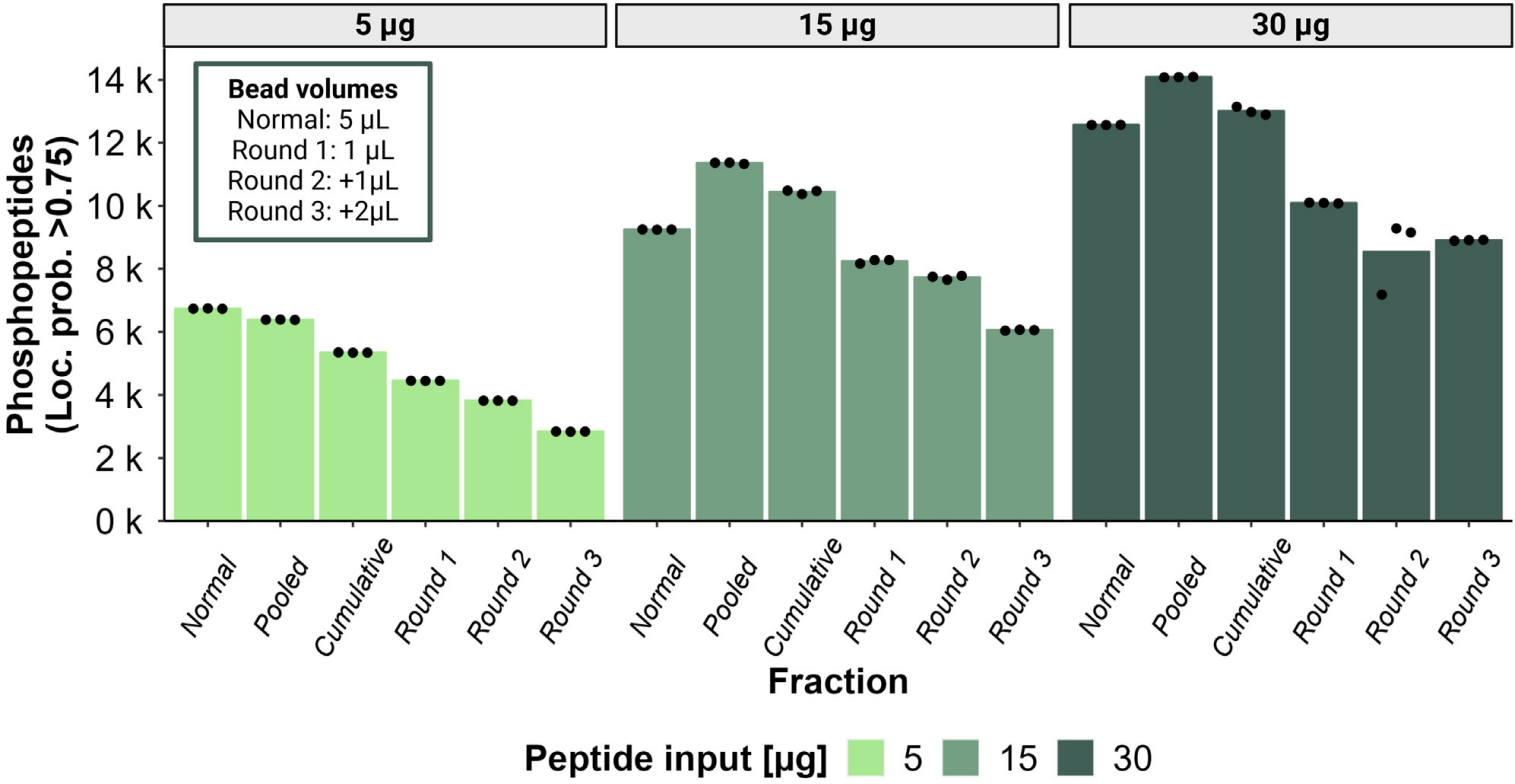
**Refined sequential enrichment pooling approach with increasing Zr-IMAC HP bead volume.** Barplots show the numbers of phosphopeptides with loc. prob. >0.75 was identified using a three-round sequential enrichment approach with increasing Zr-IMAC HP bead volume for different peptide input amounts. “Normal” represents a standard single-round enrichment with 5 μl beads. “Pooled” represents a sequential enrichment for three rounds with increasing bead volume (Round 1: 1 μl beads, Round 2: +1 μl beads, Round 3: +2 μl beads) and rounds pooled into the same elution buffer. “Round 1”, “Round 2” and “Round 3” represent the identifications in the respective separately collected and analyzed fractions. “Cumulative” refers to the cumulation of unique phosphopeptides identified in the separate fractions (“Round 1”, “Round 2”, “Round 3”) during data analysis.

Next, we tried to further exploit the benefit of pooling sequential enrichment fractions by modifying the conditions of each enrichment to favor complementary populations of phosphopeptides. In particular, we applied our observation that the amount of beads used inversely correlates with the number of multiply-phosphorylated peptides identified in a sample. We hypothesized that, when the amount of beads is limited, the competitive binding conditions lead to the favored binding of multiply-phosphorylated peptides. Consequently, to take advantage of this in a sequential enrichment strategy, we designed the following experiment: first enrichment with 1 μl of beads, second enrichment adding 1 μl of new beads, and third enrichment adding 2 μl of new beads. We either analyzed each enrichment round fraction separately and cumulated the phosphopeptides during data analysis (cumulative approach) or pooled them into one fraction by reusing the same elution buffer aliquot (pooled approach). Additionally, we performed the standard enrichment strategy with 5 μl of beads as a comparison (Fig. 6). The results revealed that there was a significant gain when using this sequential strategy. The pooled sequential approach improved the phosphoproteome depth compared to a single enrichment in standard conditions when starting with input amounts of at least 15 μg (from 9247 to 11,356 phosphopeptides for 15 μg, and from 12,568 to 14,085 phosphopeptides for 30 μg, supplemental Table S11 and supplemental File S11). Moreover, the pooled approach yielded more phosphopeptide IDs than the cumulative approach for all input amounts (6385 *versus* 5343 phosphopeptides for 5 μg, 11,356 *versus* 10,442 phosphopeptides for 15 μg and 14,085 *versus* 13,004 phosphopeptides for 30 μg). Interestingly, we observed that there was no such gain when using lower input amounts (*i.e.*, 5 μg). This might indicate that the beads-to-peptide ratio was not optimized for such low amounts and that more optimization might be required to make this strategy beneficial. Overall, we were able to confirm that sequential enrichment approaches can significantly increase phosphopeptide identifications compared to a standard one-round enrichment and observed that pooling fractions from multiple sequential enrichment rounds can outperform their separate analysis in terms of phosphopeptide identifications.

### Combination of Sequential Enrichment With LC-MS/MS analysis on an Orbitrap Astral Mass Spectrometer

Finally, the evolution of mass spectrometers is one of the most significant aspects of phosphoproteomics leading to improvements in sensitivity, speed, and depth of analysis. Therefore, we decided to complete our systematic evaluation by benchmarking the optimized phosphoproteomics workflow using the latest generation high-end proteomics-grade MS instrumentation, the Orbitrap Astral mass spectrometer.

To evaluate the performance of the Orbitrap Astral for phosphoproteomics using the optimized phosphopeptide enrichment workflow in settings best representing typical biological experiments, we performed the phosphopeptide enrichment starting from different numbers of HeLa cells. Consequently, the resulting phosphoproteome coverage reflects how sensitive the protocol is to the number of input cells. Our experiment used four replicates for six different numbers of cells, ranging from 1 million cells to 10,000 cells. The different cell samples were lysed in SDS-buffer and digested using PAC-based trypsin digestion, followed by phosphopeptide enrichment without any desalting step to minimize losses. The phosphopeptide enrichment was performed using the optimized parameters described in this project. Two rounds of sequential enrichment were performed, eluates were pooled together for analysis and evotipped samples were analyzed by a 40-SPD Whisper flow method with half-an-hour LC gradient time on the Orbitrap Astral using narrow-window data-independent acquisition (nDIA) with narrow DIA isolation windows⁠ (9).

**Fig. 7:**
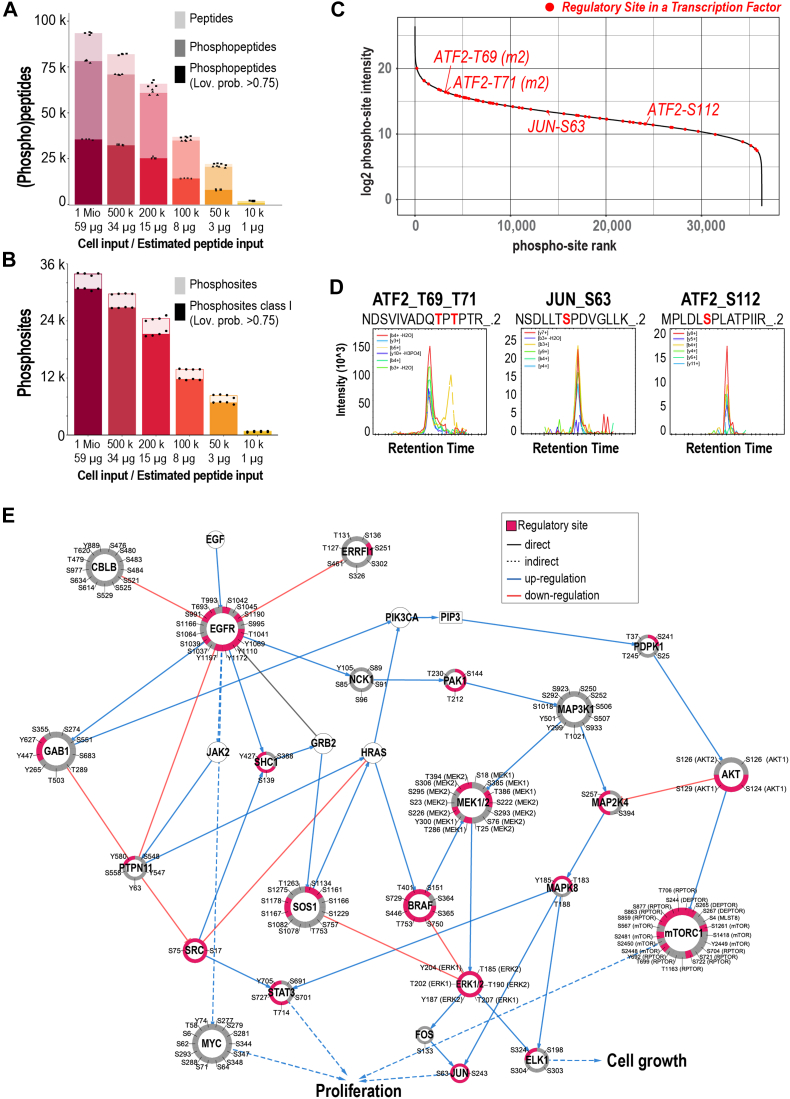
**Two-round sequential enrichment for analysis of a HeLa dilution series for LC-MS/MS analysis on an Orbitrap Astral Mass Spectrometer.***A*, barplots show the mean numbers of peptides (*light color*), phosphopeptides (*medium color*) and phosphopeptides with loc. prob. >0.75 (*dark color*) identified across four experimental replicates using different cell input amounts in a 2-round pooled sequential enrichment. Each dot represents one experimental replicate. *B*, barplots show the mean numbers of phosphosites (*light color*), and class I phosphosites (loc. prob. >0.75) (*dark color*) identified across four experimental replicates using different cell input amounts in a 2-round pooled sequential enrichment. Each dot represents one experimental replicate. *C*, rank plot based on log2 intensities of phosphosites identified in the 1 million HeLa cells phosphopeptide enrichment experiment. Known regulatory sites from transcription factors are highlighted in *red*. Representative sites plotted in panel (*D*) are labeled. *D*, Extracted Ion Chromatograms at MS2 fragment level of selected sites highlighted in panel (*C*). *E*, EGFR signaling pathway network obtained from SIGNORApp (40). The size of the nodes indicates the number of phosphosites identified in the 1 million HeLa cells phosphopeptide enrichment experiment, which are labeled in the outer ring. Each section of the outer ring (41) corresponds to a phosphorylation site. *Pink* sections indicate which phosphosite is known to be regulatory (51).

This resulting data reflects the higher sensitivity of the Orbitrap Astral MS with more than 35,000 phosphopeptides from 1 M cells or >32,000 phosphopeptides mapping to 26,000 class I phosphosites quantified in at least two samples when starting with 0.5 million cells (Fig. 7, *A* and *B*, and supplemental Tables S12 and S13; supplemental File S12). This is approximately equivalent to 34 μg of purified tryptic peptides, which is an improvement of approximately two-fold when compared to the previous coverage achieved from 30 μg of purified peptides in the Orbitrap Exploris 480 with up to 16,500 phosphopeptides (Fig. 2). The Orbitrap Astral allows a comprehensive coverage of the phosphoproteome for amounts as low as 50,000 cells (7967 phosphopeptides). With fewer cells, the output dropped below the sensitivity limits of our workflow (Fig. 7, *A* and *B* and supplemental Fig. S7, *A* and *B*). We also evaluated the site localization scores obtained from the phosphopeptides and observed a similar trend as the one observed for Orbitrap Exploris 480 data (Fig. 3*B*) with a clear drop in localization scores for lower cell inputs (supplemental Fig. S7*C*). The enrichment efficiency calculated as a function of overall intensities was >90% as expected (supplemental Fig. S7*D*).

To further evaluate whether the depth achieved in the Orbitrap Astral single-shot phosphoproteome improved the downstream functional analysis, we mapped the number of known regulatory sites for transcriptional factors contained in our data (Fig. 7*C* and supplemental File S13). We could identify relevant regulatory sites in transcription factors such as Jun or Atf2 which were distributed across the full abundance range of the obtained phosphoproteome (Fig. 7*D*). The depth of the Orbitrap Astral single-shot phosphoproteome also provided information for 2398 known kinase substrates from 277 kinases (supplemental File S13). Finally, we evaluated whether the coverage of known signaling pathways was improved in this analysis using the EGFR signaling pathway as an example. Not only could we obtain information for almost the entire network, but we could also map the most relevant regulatory sites involved in EGFR signaling (Fig. 7*E*).

**Fig. 8:**
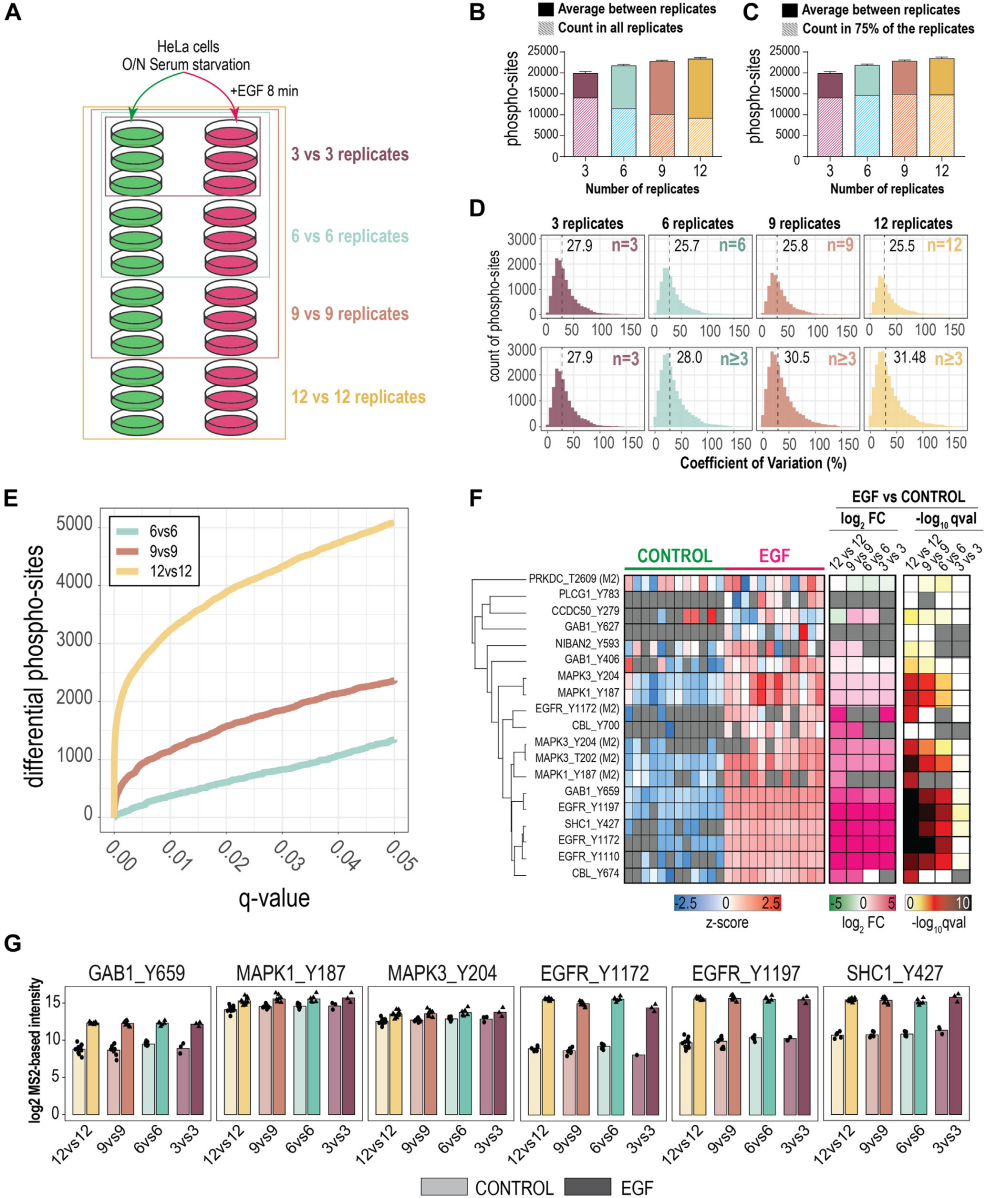
**Response to EGF treatment as a quantification benchmark of phosphoproteome analysis on an Orbitap Astral.***A*, experimental design. 12 replicates of HeLa cells treated with EGF for 8 min were compared to 12 replicates of non-treated HeLa cells. Samples were analyzed in a nested manner using either 3, 6, 9 or 12 replicates of each condition. *B*, average number of class I phosphosites identified in Spectronaut using 3, 6, 9 or 12 replicates for the search. Striped columns reflect the number of phosphosites quantified in all replicates. *C*, average number of class I phosphosites identified in Spectronaut using 3, 6, 9 or 12 replicates for the search. Striped columns reflect the number of phosphosites quantified in at least 75% of the replicates used in the analysis. *D*, histograms of the coefficient of variation at the phosphosite level between replicates measured in the different analyses. At the *top*, allowing 3 or more valid values. At the *top*, allowing only values quantified in all replicates. At the *bottom*, allowing three or more valid values. *Dashed vertical lines* indicate the median value for the coefficient of variation. *E*, number of significantly regulated phosphosites plotted against the q-value for the analysis using 6 (*light blue*), 9 (*brown*) or 12 replicates (*yellow*). *F*, heatmap of relevant sites in the EGFR signaling pathway. First heatmap shows the relative intensities (plotted as z-score per site across replicates). Second heatmap shows the log2 fold changes (EGF *versus* Control) measured in the different experimental designs: 12 *versus* 12, 9 *versus* 9, 6 *versus* 6 or 3 *versus* 3 replicates. The third heatmap shows the -log10 of the q-value obtained from the two-sample *t* test performed using 12 *versus* 12, 9 *versus* 9, 6 *versus* 6 or 3 *versus* 3 replicates. *G*, Bar plots of the mean log2 intensity of relevant sites in the EGFR signaling pathway in control (*light color*) and EGF treated samples (*dark color*).

To evaluate the quantification quality of the phosphoproteomics data derived from the Orbitrap Astral, we compared the phosphoproteome profiles of 12 replicates treated with 100 ng/ml of EGF for 8 min to activate the EGFR signaling pathway to 12 of which were used as control samples (Fig. 8*A*). With this experimental design, we first evaluated the effect of the sample size on the identification depth as well as on the completeness of the data. In this regard, we searched the data from 3, 6, 9, and 12 replicates per condition separately in Spectronaut (Fig. 8*A*). As expected, the global number of phosphosites identified by Spectronaut increased with a higher number of replicates (Fig. 8*B*), but this increase was not translated to the completeness of the dataset, since the depth of the analysis decreased when filtering for phosphosites identified in all replicates (Fig. 8*B*). When 75% of valid values were allowed, the depth of the phosphoproteome was similar regardless of the number of replicates used in the search (Fig. 8*C*). Median coefficients of variation (CVs) between the replicates were consistent independently of the number of replicates that were used in the calculation (Fig. 8*D*). This observation indicates that the full workflow, from cell culture to MS acquisition and phosphopeptide analysis, was consistent across all replicates, since introducing more replicates did not increase the variability of the measured CVs. This repeatability was also observed in a sample correlation analysis where the Pearson correlation among samples from the same condition was >0.95 (supplemental Fig. S8*A*).

Finally, we also evaluated the impact of the number of replicates in DIA-based phosphoproteomics perturbation experiments on the statistical sensitivity. Phosphosites with less than 2-thirds of valid values in at least one condition were filtered out. The data was nornalized and statistically significant phosphosites were identified using a two-samples *t* test with Benjamini-Hochberg adjusted correction for FDR control. As expected, the statistical sensitivity increased with a higher number of replicates, resulting in a significantly higher number of differentially expressed phosphosites (Fig. 8*E*). Regardless, relevant EGFR substrates and other relevant sites involved in EGFR downstream signaling (Fig. 8, *F* and *G*), were consistently regulated across the majority of the replicates analyzed, although, as expected, the statistical significance of the fold-change was more robust when using at least 6 replicates.

## Conclusion

Our extensive optimization of phosphopeptide enrichment conditions elucidated that the key parameters, including beads-to-peptide ratio, binding time, competitive-binder concentration, and sample volume, markedly influence phosphopeptide enrichment efficiency, phosphopeptide recovery, and phosphosite localization scores. Particularly, our findings underscore that multiply-phosphorylated peptides exhibit enhanced affinity towards Zr-IMAC HP beads, leading to their preferential enrichment under competitive binding conditions such as high glycolic acid concentration or low bead volumes. In line with others (44), we observed that the highest phosphoproteome coverage does not necessarily correspond to the highest enrichment efficiency and therefore recommend adapting enrichment conditions accordingly to the specific needs when aiming for either the highest phosphoproteome depth, enrichment efficiency, or proportion of multiply-phosphorylated peptides.

Throughout our investigation of enrichment efficiency, we found that enrichment efficiency calculated from abundance exhibited consistently higher values than that calculated from peptide counts. A potential explanation for the comparably low enrichment efficiency based on counts in our DIA-based study in contrast to other data-dependent acquisition (DDA) based studies reporting values >90% (13, 17, 22) could be that DIA provides higher peptide identification rates than DDA (45, 46, 47), increasing the detection of low-abundant non-phosphorylated peptides and possibly biasing count-based enrichment efficiency towards lower values than expected. Enrichment efficiency based on abundance could therefore serve as a potentially more nuanced representation of peptide *versus* phosphopeptide ratio in phosphoproteomics samples, especially in DIA-based studies.

We propose sequential phosphopeptide enrichment as a powerful strategy to further amplify the depth of phosphopeptide analysis. Our study indicates that while initial enrichment rounds demonstrate higher enrichment efficiency, subsequent rounds do not, providing only minimal improvements in phosphoproteome depth after the third round. Therefore, a sequential approach with two enrichment rounds seems to be favorable for most applications in a range from 20 μg to 2.5 μg of peptide input, although more rounds might offer further improvement for high peptide input amounts.

Importantly, the post-acquisition analysis in the search engine (*i.e.*, Spectronaut) of separate fractions or experimental conditions has so far been the standard method for method optimization. However, in the case of sequential enrichment, the data analysis of one phosphopeptide entity with multiple LFQ intensities derived from independent enrichments is not trivial. It can result in higher variability and/or increased CVs, hindering subsequent data interpretation, especially when performing label-free quantification. Our data shows that pooling fractions into a single LC-MS/MS analysis is a good alternative to circumvent these issues while decreasing the LC-MS/MS analysis time. In this regard, we present an improved strategy based on the incremental addition of beads and subsequent fraction pooling, which offers up to 20% boosted phosphoproteome coverage compared to standard enrichment, while maintaining high sample-throughput and straightforward data analysis.

Finally, we report that our optimized phosphoproteomics pipeline can be translated to the newest generation of mass spectrometers, such as the Orbitrap Astral, that can increase the phosphopeptide coverage by 2-fold, allowing for deep phosphoproteomics analysis without the need to scale up the starting cell amounts. Evaluation of the repeatability of our optimized workflow in combination with data acquisition on the Orbitrap Astral indicated that the full workflow, from cell culture to MS acquisition and phosphopeptide analysis, yielded repeatable deep phosphoproteomes across all replicates. Moreover, we observed that the regulation of many phosphosites involved in relevant signaling pathways can be already measured using only three biological replicates, although at least six replicates are recommended for statistical analysis. Future research might explore the refinement of enrichment conditions that simultaneously maximize both enrichment efficiency and phosphoproteome depth. Furthermore, as bead type/composition (13, 22, 48), bead metal purity (43) and affinity material format (49) have also been shown to influence phosphopeptide yield, enrichment efficiency, and multiplicity of enriched phosphopeptides, further comparison and optimization of the beads themselves in terms of capacity and availability of binding sites could potentially further improve phosphopeptide enrichment. Combining optimal enrichment conditions with strategic application-tailored pooling of sequential enrichments could pave the way for more comprehensive phosphoproteomics investigations, allowing this workflow to be translated to relevant applications, such as single-spheroid analysis for high-throughput drug screening (12) or *in vivo* signaling using tissues (50). Although more specific applications might require further optimization, this work can serve as a roadmap for sensitive phosphoproteomics analysis.

## Data Availability

The mass spectrometry proteomics data and supplemental files have been deposited to the ProteomeXchange Consortium *via* the PRIDE partner repository with the dataset identifier PXD045601.

## Supplemental Data

This article contains supplemental data.

## Conflict of interest

The authors declare that they have no conflicts of interest with the contents of this article.

## References

[1] Humphrey S.J., Karayel O., James D.E., Mann M. (2018). High-throughput and high-sensitivity phosphoproteomics with the EasyPhos platform. Nat. Protoc..

[2] Olsen J.V., Mann M. (2013). Status of large-scale analysis of post-translational modifications by mass spectrometry. Mol. Cell. Proteomics.

[3] Lundby A., Andersen M.N., Steffensen A.B., Horn H., Kelstrup C.D., Francavilla C. (2013). *In vivo* phosphoproteomics analysis reveals the cardiac targets of β-adrenergic receptor signaling. Sci. Signal..

[4] Villén J., Beausoleil S.A., Gerber S.A., Gygi S.P. (2007). Large-scale phosphorylation analysis of mouse liver. PNAS.

[5] Olsen J.V., Blagoev B., Gnad F., Macek B., Kumar C., Mortensen P. (2006). Global, *in vivo*, and site-specific phosphorylation dynamics in signaling networks. Cell.

[6] Ochoa D., Jarnuczak A.F., Viéitez C., Gehre M., Soucheray M., Mateus A. (2020). The functional landscape of the human phosphoproteome. Nat. Biotechnol..

[7] Sharma K., D'Souza R.C.J., Tyanova S., Schaab C., Wiśniewski J.R., Cox J. (2014). Ultradeep human phosphoproteome reveals a distinct regulatory nature of Tyr and Ser/Thr-based signaling. Cell Rep..

[8] Bekker-Jensen D.B., Kelstrup C.D., Batth T.S., Larsen S.C., Haldrup C., Bramsen J.B. (2017). An optimized shotgun strategy for the rapid generation of comprehensive human proteomes. Cell Syst..

[9] Guzman U.H., Martinez-Val A., Ye Z., Damoc E., Arrey T.N., Pashkova A. (2024). Ultra-fast label-free quantification and comprehensive proteome coverage with narrow-window data-independent acquisition. Nat. Biotechnol..

[10] Wu R., Haas W., Dephoure N., Huttlin E.L., Zhai B., Sowa M.E. (2011). A large-scale method to measure absolute protein phosphorylation stoichiometries. Nat. Methods.

[11] Yang S., Han Y., Li Y., Zhang L., Yan G., Yuan J. (2023). Rapid and high-sensitive phosphoproteomics elucidated the spatial dynamics of the mouse brain. Anal. Chem..

[12] Martínez-Val A., Fort K., Koenig C., van der Hoeven L., Franciosa G., Moehring T. (2023). Hybrid-DIA: intelligent data acquisition integrates targeted and discovery proteomics to analyze phospho-signaling in single spheroids. Nat. Commun..

[13] Leutert M., Rodríguez-Mias R.A., Fukuda N.K., Villén J. (2019). R2-P2 rapid-robotic phosphoproteomics enables multidimensional cell signaling studies. Mol. Syst. Biol..

[14] Tape C.J., Worboys J.D., Sinclair J., Gourlay R., Vogt J., McMahon K.M. (2014). Reproducible automated phosphopeptide enrichment using magnetic TiO2 and Ti-IMAC. Anal. Chem..

[15] Koenig C., Martinez-Val A., Naicker P., Stoychev S., Jordaan J., Olsen J.V. (2023). Protocol for high-throughput semi-automated label-free- or TMT-based phosphoproteome profiling. STAR Protoc..

[16] Emdal K.B., Palacio-Escat N., Wigerup C., Eguchi A., Nilsson H., Bekker-Jensen D.B. (2022). Phosphoproteomics of primary AML patient samples reveals rationale for AKT combination therapy and p53 context to overcome selinexor resistance. Cell Rep..

[17] Chen C.-W., Tsai C.-F., Lin M.-H., Lin S.-Y., Hsu C.-C. (2023). Suspension trapping-based sample preparation workflow for in-depth plant phosphoproteomics. Anal. Chem..

[18] Zhou H., Ye M., Dong J., Corradini E., Cristobal A., Heck A.J.R. (2013). Robust phosphoproteome enrichment using monodisperse microsphere-based immobilized titanium (IV) ion affinity chromatography. Nat. Protoc..

[19] Gates M.B., Tomer K.B., Deterding L.J. (2010). Comparison of metal and metal oxide media for phosphopeptide enrichment prior to mass spectrometric analyses. J. Am. Soc. Mass Spectrom..

[20] Leitner A. (2010). Phosphopeptide enrichment using metal oxide affinity chromatography. Trends Anal. Chem..

[21] Li Q., Ning Z., Tang J., Nie S., Zeng R. (2009). Effect of peptide-to-TiO2 beads ratio on phosphopeptide enrichment selectivity. J. Proteome Res..

[22] Arribas Diez I., Govender I., Naicker P., Stoychev S., Jordaan J., Jensen O.N. (2021). Zirconium(IV)-IMAC revisited: improved performance and phosphoproteome coverage by magnetic microparticles for phosphopeptide affinity enrichment. J. Proteome Res..

[23] Jensen S.S., Larsen M.R. (2007). Evaluation of the impact of some experimental procedures on different phosphopeptide enrichment techniques. Rapid Commun. Mass Spectrom..

[24] Li J., Wang J., Yan Y., Li N., Qing X., Tuerxun A. (2022). Comprehensive evaluation of different TiO2-based phosphopeptide enrichment and fractionation methods for phosphoproteomics. Cells.

[25] Palmisano G., Lendal S.E., Larsen M.R. (2011). Titanium dioxide enrichment of sialic acid-containing glycopeptides. Methods Mol. Biol..

[26] Palmisano G., Lendal S.E., Engholm-Keller K., Leth-Larsen R., Parker B.L., Larsen M.R. (2010). Selective enrichment of sialic acid-containing glycopeptides using titanium dioxide chromatography with analysis by HILIC and mass spectrometry. Nat. Protoc..

[27] Larsen M.R., Thingholm T.E., Jensen O.N., Roepstorff P., Jørgensen T.J.D. (2005). Highly selective enrichment of phosphorylated peptides from peptide mixtures using titanium dioxide microcolumns. Mol. Cell. Proteomics.

[28] Thingholm T.E., Jensen O.N., Robinson P.J., Larsen M.R. (2008). SIMAC (sequential elution from IMAC), a phosphoproteomics strategy for the rapid separation of monophosphorylated from multiply phosphorylated peptides. Mol. Cell. Proteomics.

[29] Tsai C.-F., Hsu C.-C., Hung J.-N., Wang Y.-T., Choong W.-K., Zeng M.-Y. (2014). Sequential phosphoproteomic enrichment through complementary metal-directed immobilized metal ion affinity chromatography. Anal. Chem..

[30] Bekker-Jensen D.B., Bernhardt O.M., Hogrebe A., Martinez-Val A., Verbeke L., Gandhi T. (2020). Rapid and site-specific deep phosphoproteome profiling by data-independent acquisition without the need for spectral libraries. Nat. Commun..

[31] Lou R., Liu W., Li R., Li S., He X., Shui W. (2021). DeepPhospho accelerates DIA phosphoproteome profiling through in silico library generation. Nat. Commun..

[32] Batth T.S., Tollenaere M.X., Rüther P., Gonzalez-Franquesa A., Prabhakar B.S., Bekker-Jensen S. (2019). Protein aggregation capture on microparticles enables multipurpose proteomics sample preparation. Mol. Cell. Proteomics.

[33] Bekker-Jensen D.B., Martínez-Val A., Steigerwald S., Rüther P., Fort K.L., Arrey T.N. (2020). A compact quadrupole-orbitrap mass spectrometer with FAIMS interface improves proteome coverage in short LC gradients. Mol. Cell. Proteomics.

[34] Reiter L., Rinner O., Picotti P., Hüttenhain R., Beck M., Brusniak M.-Y. (2011). mProphet: automated data processing and statistical validation for large-scale SRM experiments. Nat. Methods.

[35] Martinez-Val A., Bekker-Jensen D.B., Hogrebe A., Olsen J.V. (2021). Data processing and analysis for DIA-based phosphoproteomics using Spectronaut. Methods Mol. Biol..

[36] Gu Z., Eils R., Schlesner M. (2016). Complex heatmaps reveal patterns and correlations in multidimensional genomic data. Bioinformatics.

[37] Wickham H. (2016).

[38] Wickham H., Averick M., Bryan J., Chang W., McGowan L., François R. (2019). Welcome to the tidyverse. J. Open Source Softw..

[39] Shannon P., Markiel A., Ozier O., Baliga N.S., Wang J.T., Ramage D. (2003). A software environment for integrated models of biomolecular interaction networks. Genome Res..

[40] Marinis I.D., Lo Surdo P., Cesareni G., Perfetto L. (2022). SIGNORApp: a Cytoscape 3 application to access SIGNOR data. Bioinformatics.

[41] Legeay M., Doncheva N.T., Morris J.H., Jensen L.J. (2020). Visualize omics data on networks with Omics visualizer, a Cytoscape app. F1000Res..

[42] Palmisano G., Parker B.L., Engholm-Keller K., Lendal S.E., Kulej K., Schulz M. (2012). A novel method for the simultaneous enrichment, identification, and quantification of phosphopeptides and sialylated glycopeptides applied to a temporal profile of mouse brain development. Mol. Cell. Proteomics.

[43] Ye J., Zhang X., Young C., Zhao X., Hao Q., Cheng L. (2010). Optimized IMAC-IMAC protocol for phosphopeptide recovery from complex biological samples. J. Proteome Res..

[44] Oliinyk D., Will A., Schneidmadel F.R., Humphrey S.J., Meier F. (2023). μPhos: a scalable and sensitive platform for functional phosphoproteomics. bioRxiv.

[45] Bruderer R., Bernhardt O.M., Gandhi T., Xuan Y., Sondermann J., Schmidt M. (2017). Optimization of experimental parameters in data-independent mass spectrometry significantly increases depth and reproducibility of results. Mol. Cell. Proteomics.

[46] Michalski A., Cox J., Mann M. (2011). More than 100,000 detectable peptide species elute in single shotgun proteomics runs but the majority is inaccessible to data-dependent LC-MS/MS. J. Proteome Res..

[47] Steger M., Demichev V., Backman M., Ohmayer U., Ihmor P., Müller S. (2021). Time-resolved *in vivo* ubiquitinome profiling by DIA-MS reveals USP7 targets on a proteome-wide scale. Nat. Commun..

[48] Post H., Penning R., Fitzpatrick M.A., Garrigues L.B., Wu W., MacGillavry H.D. (2017). Robust, sensitive, and automated phosphopeptide enrichment optimized for low sample amounts applied to primary hippocampal neurons. J. Proteome Res..

[49] Ruprecht B., Koch H., Medard G., Mundt M., Kuster B., Lemeer S. (2015). Comprehensive and reproducible phosphopeptide enrichment using iron immobilized metal ion affinity chromatography (Fe-IMAC) columns. Mol. Cell. Proteomics.

[50] Snieckute G., Ryder L., Vind A.C., Wu Z., Arendrup F.S., Stoneley M. (2023). ROS-induced ribosome impairment underlies ZAKα-mediated metabolic decline in obesity and aging. Science.

[51] Hornbeck P.V., Zhang B., Murray B., Kornhauser J.M., Latham V., Skrzypek E. (2015). PhosphoSitePlus, 2014: mutations, PTMs and recalibrations. Nucleic Acids Res..

