# Supplementary material for: Systematic Optimization of Automated Phosphopeptide Enrichment for High-Sensitivity Phosphoproteomics
Source: Mol Cell Proteomics. 2024 Mar 27;23(5):100754. doi: 10.1016/j.mcpro.2024.100754 (PMC11087715; doi:10.1016/j.mcpro.2024.100754)
Supplement: Supplemental Figures S1–S8 and Tables S1–S13 [file mmc1.pdf]

Supplementary Material for:

Systematic optimization of automated phosphopeptide enrichment for high-sensitivity phosphoproteomics

Patricia Bortel [1,2], Ilaria Piga [3], Claire Koenig [3], Christopher Gerner [1,4], Ana Martinez-Val [3,5*], Jesper V. Olsen [3*].

[1] Department of Analytical Chemistry, Faculty of Chemistry, University of Vienna, Waehringer Str. 38, 1090 Vienna, Austria

[2] Vienna Doctoral School in Chemistry (DoSChem), University of Vienna, Waehringer Str. 42, 1090 Vienna, Austria

[3] Novo Nordisk Foundation Center for Protein Research, Proteomics Program, Faculty of Health and Medical Sciences, University of Copenhagen, Copenhagen, Denmark

[4] Joint Metabolome Facility, University of Vienna and Medical University of Vienna, Waehringer Str. 38, 1090 Vienna, Austria.

[5] Current address: Centro Nacional de Investigaciones Cardiovasculares Carlos III (CNIC), Madrid 28029, Spain.

* correspondence to ana.martinezdelval@cnic.es; jesper.olsen@cpr.ku.dk

Supplementary figures

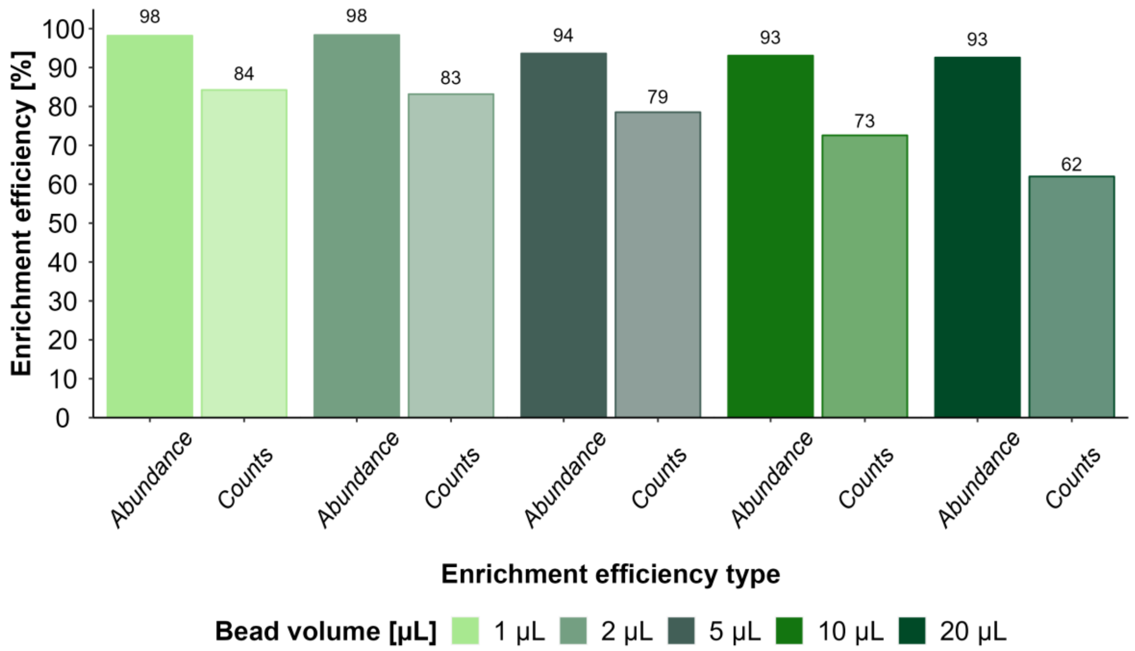

Supplementary Figure S1. Enrichment efficiency based on counts vs. enrichment efficiency based on abundance for samples of the bead volume evaluation experiment.

Barplots show the enrichment efficiency calculated from counts (number of phosphopeptides / total number of peptides) and the enrichment efficiency calculated from abundance (MS signal from phosphopeptides vs. total signal) for samples for which different bead volumes were used during phosphopeptide enrichment.

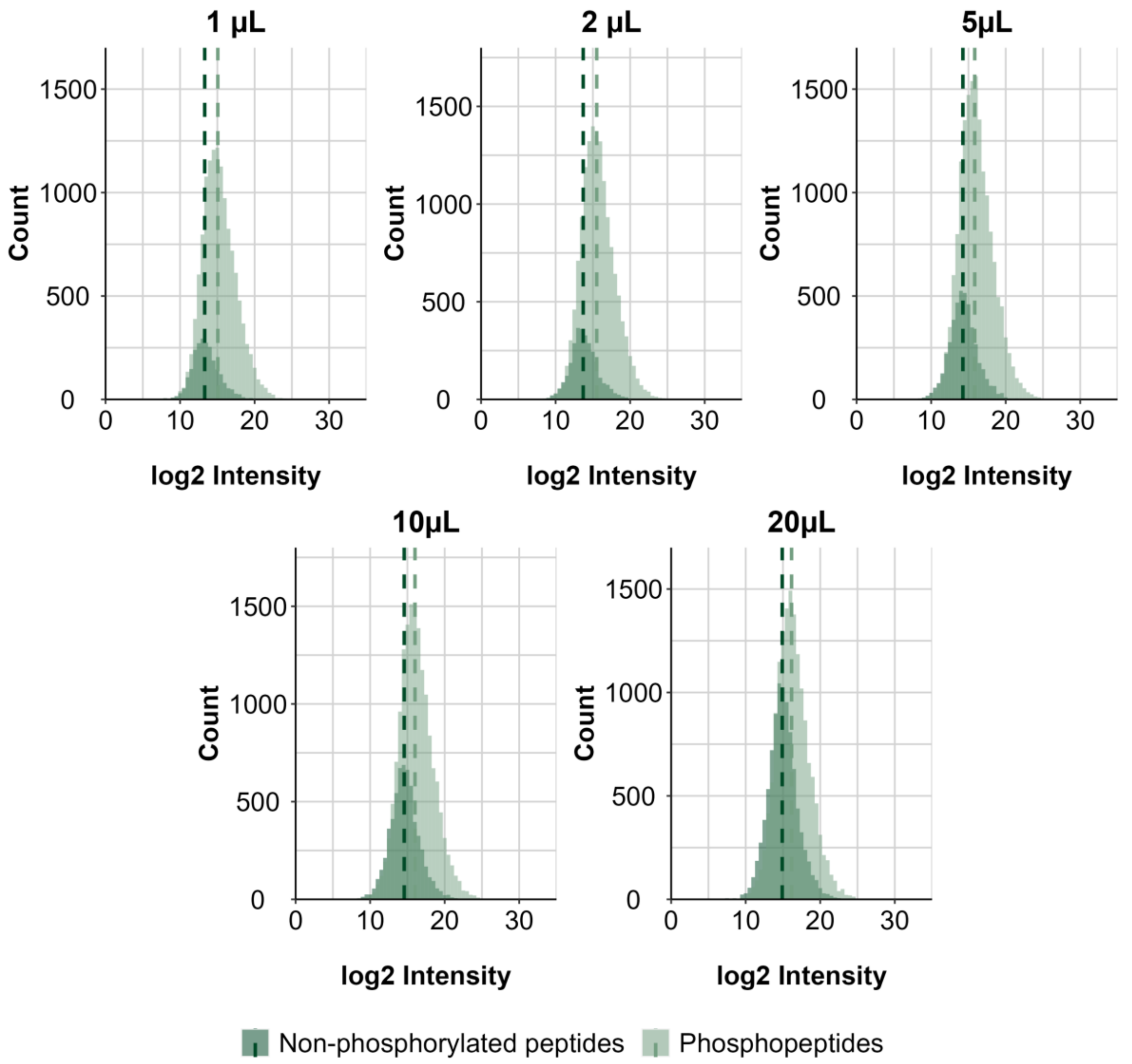

Supplementary Figure S2. Intensity distribution of non-phosphorylated peptides and phosphopeptides of the bead volume evaluation experiment.

Histograms show the distribution of mean log2 intensities between three experimental replicates of non-phosphorylated peptides (dark green color) and phosphopeptides (light green color) using different bead volumes for phosphopeptide enrichment. Dashed lines represent the median intensities of each cohort.

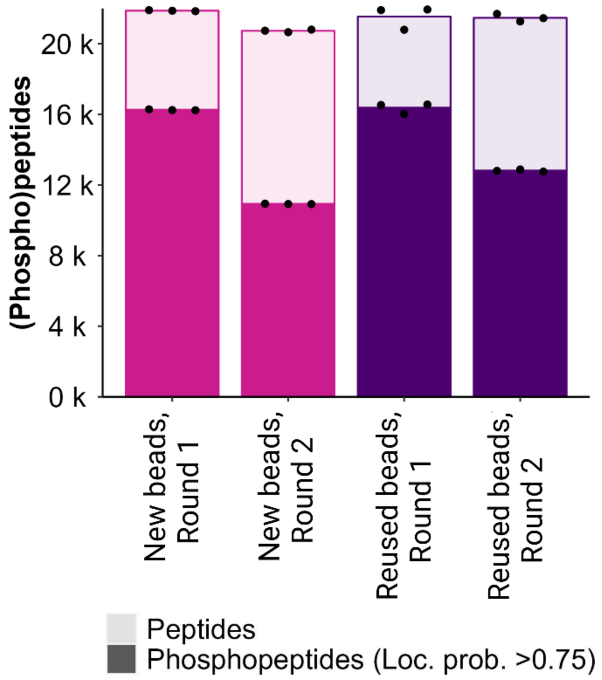

Supplementary Figure S3. Effect of bead reuse or exchange in a sequential phosphopeptide enrichment approach.

Barplots show the mean numbers of peptides (light color) or phosphopeptides with loc. prob. >0.75 (dark color) identified across three experimental replicates in a sequential enrichment approach with two rounds in which the beads were either exchanged after the first round (pink) or reused (purple). The peptide input amount for the enrichment was 30 µg for all conditions. Each dot indicates one experimental replicate.

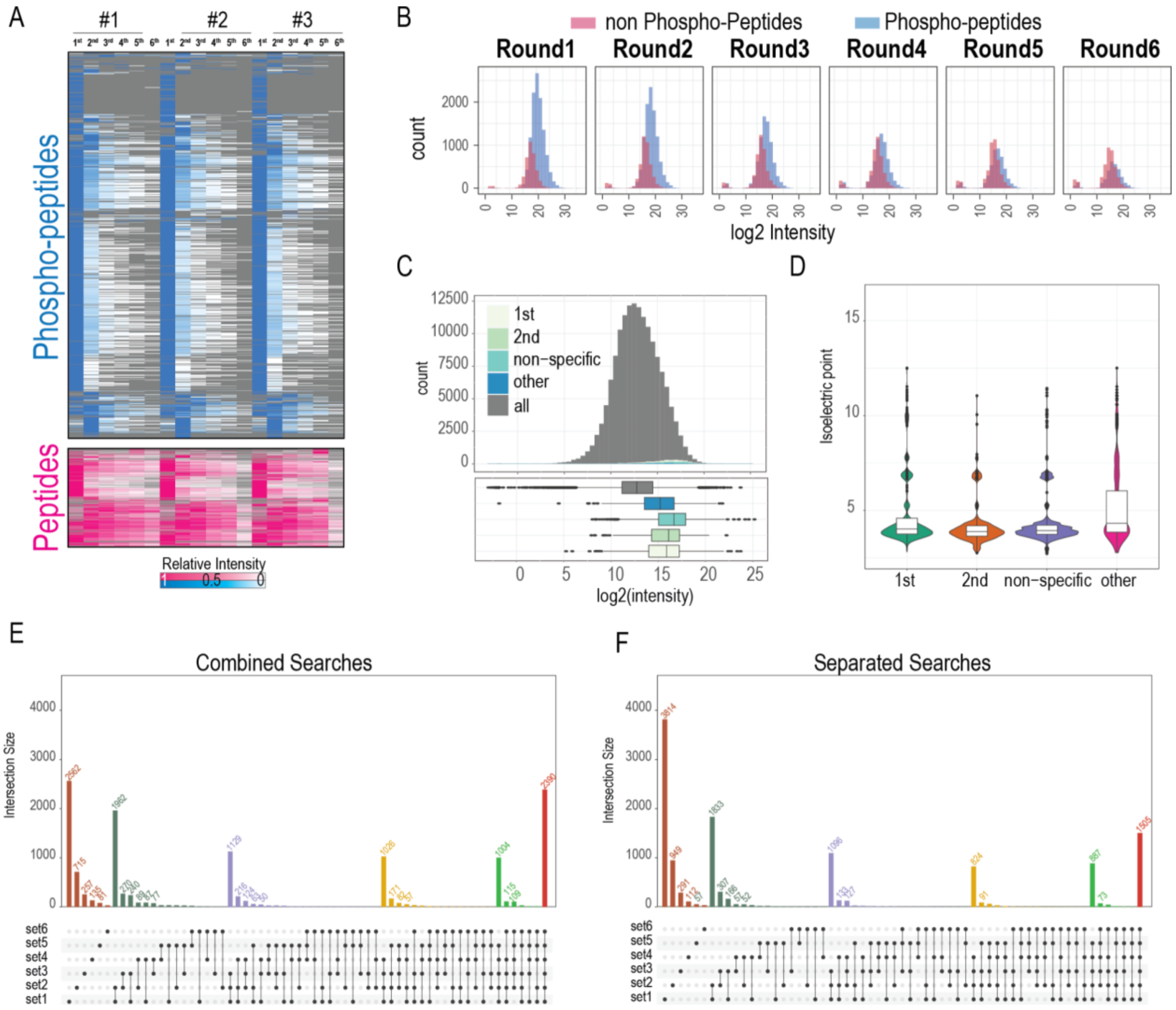

Supplementary Figure S4. (Phospho)peptide elution profiles across fractions upon sequential enrichment with 6 rounds.

(A) Elution profile of phosphopeptides (blue) and non-phospho peptides (pink) across six sequential enrichments using 20 µg of peptide input in three separate experimental replicates. The intensity plotted is scaled from 1 to 0 across the six sequential enrichments. **(B)** Histograms showing the peptide intensity (in log2) distribution for one experiment and six sequential enrichments. In blue: phosphopeptides, in pink: non-phospho peptides. **(C)** Histogram (top) and boxplot (bottom) showing the whole peptide intensity distribution (gray) of the whole proteome of A549 (analyzed as a single-shot in Orbitrap-Astral, data from Guzman et al[9]). highlighted in blue colors, the distribution of the non-phosphorylated peptide intensities found in the six sequential enrichment experiments when measured in a whole proteome. The different categories (1st, 2nd, non-specific and other) correspond to different elution profiles of the non-phosphopeptides as observed in panel A. 1st: peptides eluting mainly in the 1st enrichment. 2nd: peptides eluting mainly in the second enrichment. Non-specific: peptides showing a constant elution across the first enrichments. Other: other peptides with no pattern in their elution. **(D)** Isoelectric point distribution values in the non-phosphopeptides measured in the six enrichment experiments shown in panel A. Categories are the same as described in panel C. **(E-F)** Effect of search strategy on the sequential enrichment results. **(E)** Results of overlap in identifications between enrichments when the

samples are analyzed together in Spectronaut. **(F)** Results of overlap in identifications between enrichments when the samples are analyzed separately in Spectronaut.

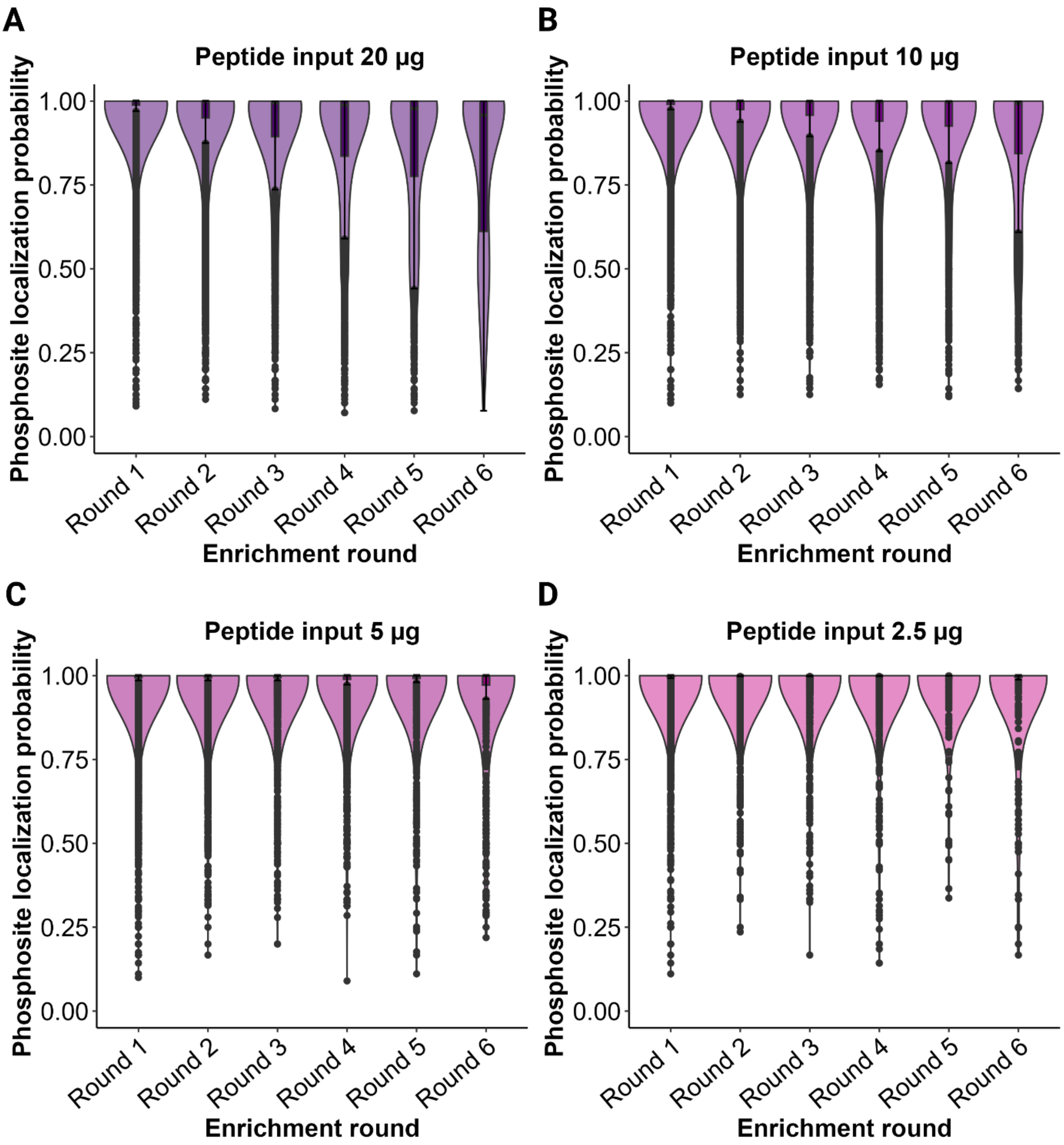

Supplementary Figure S5. Phosphosite localization in sequential enrichment approach with 6 rounds.

(A-D) Violin plots show the range and distribution of the localization probability of phosphosites identified in each enrichment round of a 6 round sequential enrichment, using different peptide input amounts (A: 20 µg, B: 10 µg, C: 5 µg, D: 2.5 µg)

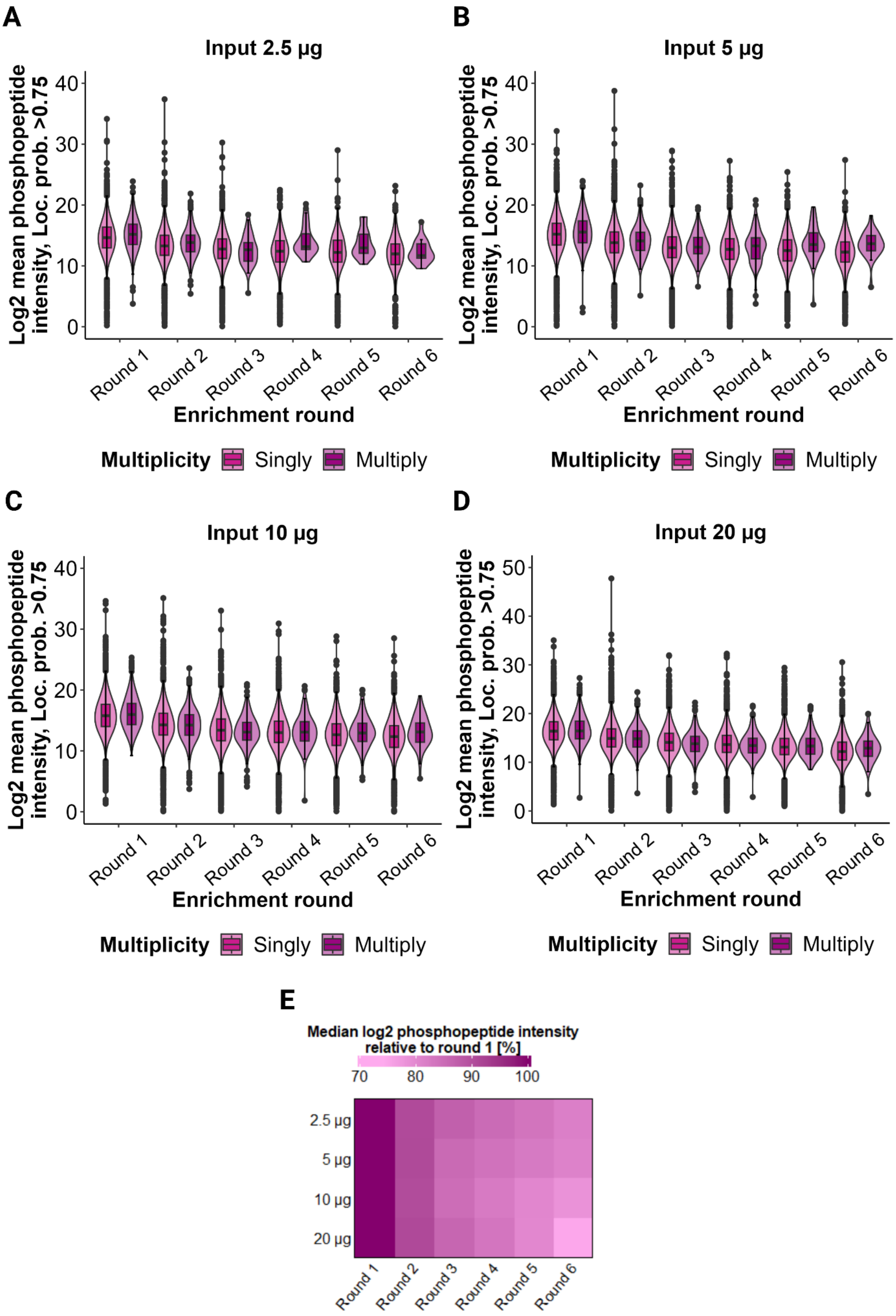

A
Input 2.5 µg
B
Input 5 µg
C
Input 10 µg
D
Input 20 µg
Log2 mean phosphopeptide intensity, Loc. prob. >0.75
Round 1
Round 2
Round 3
Round 4
Round 5
Round 6
Enrichment round
Multiplicity
Singly
Multiply
E
Median log2 phosphopeptide intensity relative to round 1 [%]
70
80
90
100
2.5 µg
5 µg
10 µg
20 µg

Supplementary Figure S6. Phosphopeptide IDs in a sequential enrichment approach with 3 rounds and different bead-binding times.

(A-D) Violin plots show the distribution log2 mean phosphopeptide intensities of singly and multiply phosphorylated peptides in each enrichment round of a 6 round sequential enrichment, using different peptide input amounts (A: 20 µg, B: 10 µg, C: 5 µg, D: 2.5 µg).

(**E**) Heatmap shows the median log 2 phosphopeptide intensities of singly and multiply phosphorylated peptides relative to round 1 of a 6 round sequential enrichment, using different peptide input amounts.

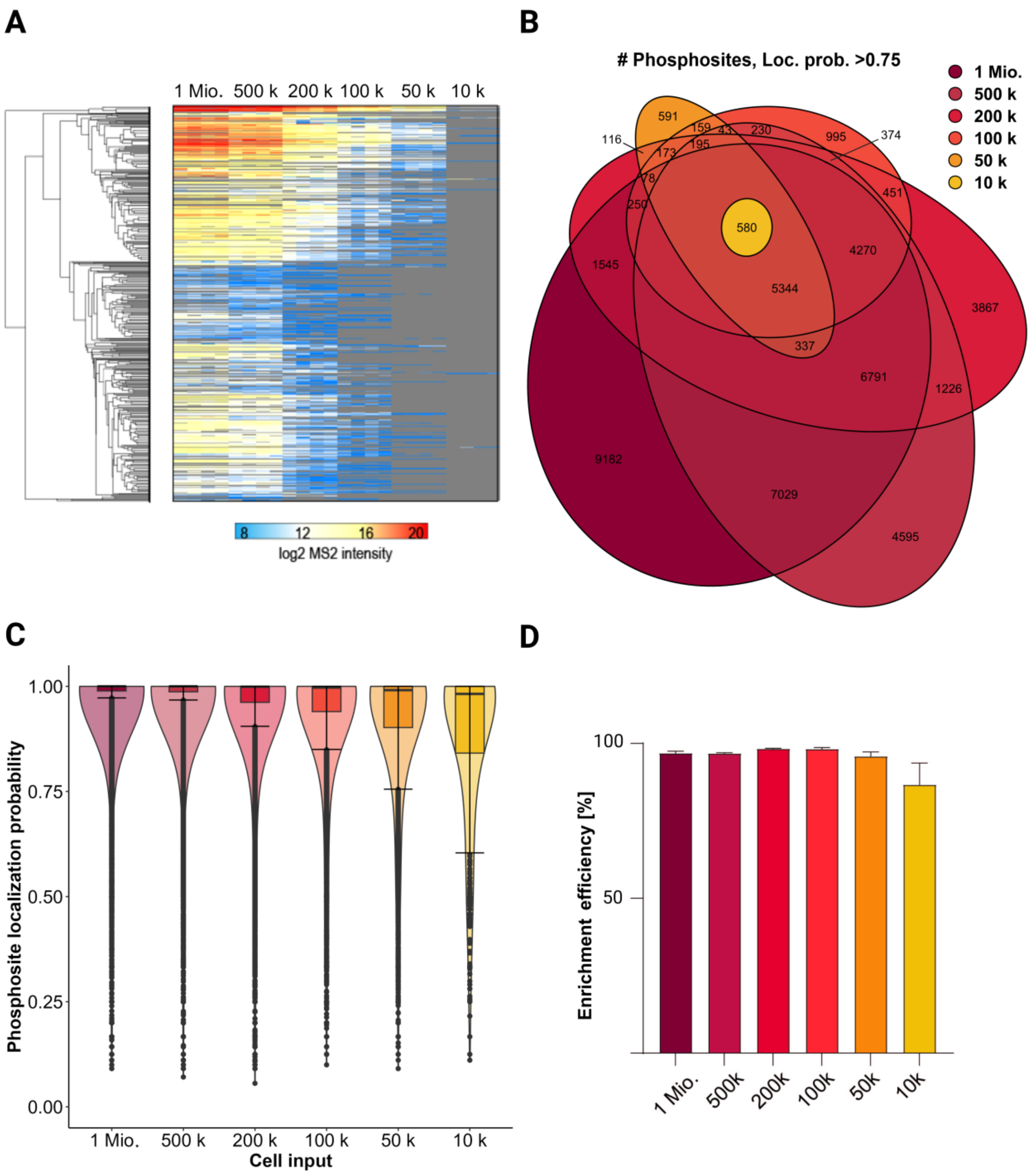

Supplementary Figure S7. Detailed assessment of a 2-round sequential enrichment pooled approach for analysis of a HeLa dilution series in combination with LC-MS/MS analysis on an Orbitrap Astral Mass Spectrometer.

(A) Heatmap shows the log2 mean intensities of unique phosphopeptides identified across four experimental replicates using different cell input amounts in a 2-round pooled sequential enrichment. **(B)** The venn diagram shows uniquely and commonly identified phosphosites with loc. prob. >0.75 among different cell input amounts in a 2-round pooled sequential enrichment. **(C)** Violin plots show the range and distribution of the localization probability of phosphosites identified using different cell input amounts. **(D)** Barplots show the average of the phosphopeptide enrichment efficiency (based on MS intensity) of data obtained using the Orbitrap Astral with different cell amounts as input in the workflow.

A

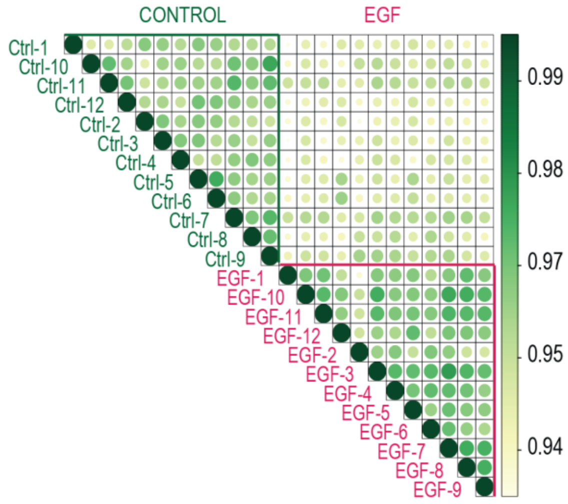

B

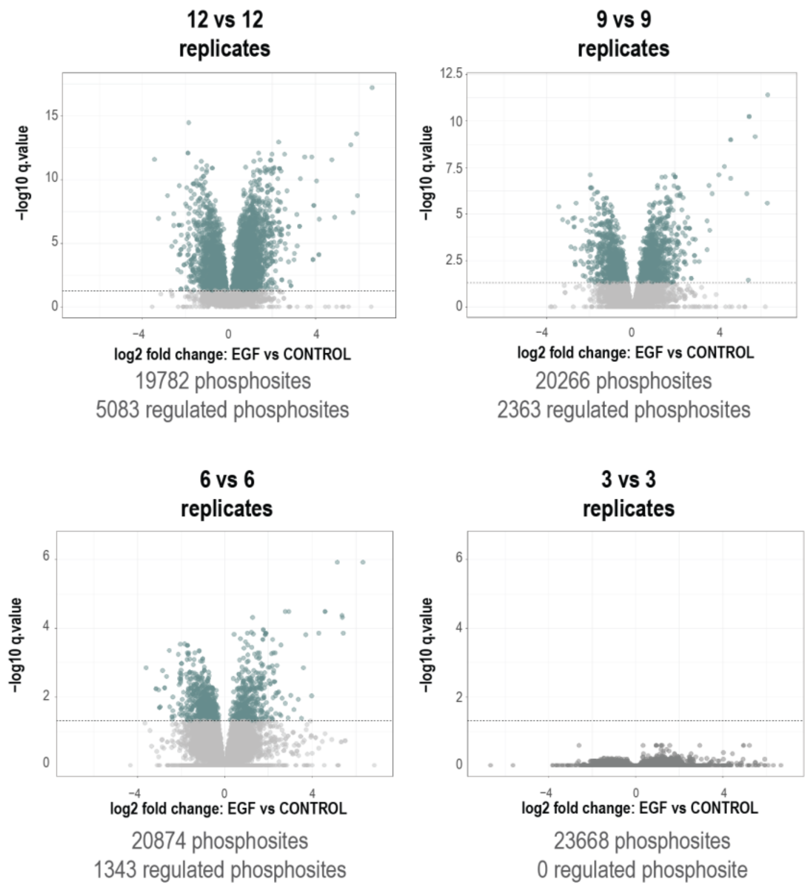

Supplementary Figure S8. Quantification benchmark of our phosphoproteomics workflow in combination with data acquisition on an Orbitrap Astral mass spectrometer.

(A) Correlation plot between biological replicates of HeLa cells after overnight serum starvation (Control) and after treatment with EGF (EGF). Color gradient (light yellow to dark green) and dot size indicate the value for the Pearson correlation (from 0.94 to 1) between

replicates. **(B)** Volcano plots for the statistical test (two-sample t-test, Benjamini Hochberg FDR) comparing different numbers of replicates (12, 9, 6 or 3) in the EGF versus Control experiment. Horizontal dashed line indicates a q-value cutoff of 0.05. Colored points indicate statistically significantly regulated phosphosites.

Supplementary tables

Zr-IMAC HP bead volume [µL]	Cutoff	IDs
1	Peptides	2428
2	Peptides	2996
5	Peptides	4432
10	Peptides	6010
20	Peptides	9213
1	Phosphopeptides (Loc. prob. >0.75)	12962
2	Phosphopeptides (Loc. prob. >0.75)	14786
5	Phosphopeptides (Loc. prob. >0.75)	16193
10	Phosphopeptides (Loc. prob. >0.75)	15895
20	Phosphopeptides (Loc. prob. >0.75)	15026

Supplementary Table S1. Mean peptide and phosphopeptide (Loc. prob. >0.75) IDs identified across three experimental replicates using different proportions of Zr-IMAC HP bead volumes. The peptide input amount for the enrichment was 30 µg for all conditions.

Bead binding time [min]	Cutoff	IDs
5	Peptides	4614
10	Peptides	5188
20	Peptides	5549
30	Peptides	6115
5	Phosphopeptides (Loc. prob. >0.75)	15700
10	Phosphopeptides (Loc. prob. >0.75)	15516
20	Phosphopeptides (Loc. prob. >0.75)	16440
30	Phosphopeptides (Loc. prob. >0.75)	16046

Supplementary Table S2. Mean peptide and phosphopeptide (Loc. prob. >0.75) IDs identified across three experimental replicates using different sample-bead binding times. The peptide input amount for the enrichment was 30 µg for all conditions.

Glycolic acid [M]	Cutoff	IDs
0	Peptides	5714
0.1	Peptides	4483
0.5	Peptides	3052
1	Peptides	2425
2	Peptides	1773
0	Phosphopeptides (Loc. prob. >0.75)	16342
0.1	Phosphopeptides (Loc. prob. >0.75)	15929
0.5	Phosphopeptides (Loc. prob. >0.75)	15292
1	Phosphopeptides (Loc. prob. >0.75)	14001
2	Phosphopeptides (Loc. prob. >0.75)	10811

Supplementary Table S3. Mean peptide and phosphopeptide (Loc. prob. >0.75) IDs identified across three experimental replicates using different proportions of glycolic acid in the loading buffer. The peptide input amount for the enrichment was 30 µg for all conditions.

Ammonium hydroxide [%]	Cutoff	IDs
0.1	Peptides	3987
0.5	Peptides	3786
1	Peptides	4301
2	Peptides	4408
0.1	Phosphopeptides (Loc. prob. >0.75)	16071
0.5	Phosphopeptides (Loc. prob. >0.75)	16516
1	Phosphopeptides (Loc. prob. >0.75)	16515
2	Phosphopeptides (Loc. prob. >0.75)	15982

Supplementary Table S4. Mean peptide and phosphopeptide (Loc. prob. >0.75) IDs identified across three experimental replicates using different proportions of ammonium hydroxide in the elution buffer. The peptide input amount for the enrichment was 30 µg for all conditions.

Peptide input amount [µg]	Cutoff	IDs
5	Peptides	1515
15	Peptides	4312
30	Peptides	3068
5	Phosphopeptides (Loc. prob. >0.75)	6888
15	Phosphopeptides (Loc. prob. >0.75)	10755
30	Phosphopeptides (Loc. prob. >0.75)	12480

Supplementary Table S5. Mean peptide and phosphopeptide (Loc. prob. >0.75) IDs identified across three experimental replicates using different peptide input amounts.

Sample volume [µL]	Loading buffer volume [µL]	Cutoff	IDs
7.5	200	Peptides	2691
30	200	Peptides	2392
60	200	Peptides	2361
120	200	Peptides	2258
7.5	200	Phosphopeptides (Loc. prob. >0.75)	16132
30	200	Phosphopeptides (Loc. prob. >0.75)	15218
60	200	Phosphopeptides (Loc. prob. >0.75)	14585
120	200	Phosphopeptides (Loc. prob. >0.75)	13770

Supplementary Table S6. Mean peptide and phosphopeptide (Loc. prob. >0.75) IDs identified across three experimental replicates using the same peptide input amount (30 µg) diluted in different sample volumes, mixed with the same volume of loading buffer (200 µL).

Sample volume [µL]	Loading buffer volume [µL]	Cutoff	IDs
15	100	Peptides	3033
15	200	Peptides	1826
15	300	Peptides	1384
15	100	Peptides	1238
15	100	Phosphopeptides (Loc. prob. >0.75)	15327
15	200	Phosphopeptides (Loc. prob. >0.75)	15109
15	300	Phosphopeptides (Loc. prob. >0.75)	14925
15	400	Phosphopeptides (Loc. prob. >0.75)	15294

Supplementary Table S7. Mean peptide and phosphopeptide (Loc. prob. >0.75) IDs identified across three experimental replicates using the same peptide input amount (30 µg) diluted in the same sample volume (15 µL), mixed with different volumes of loading buffer.

Sequential bead approach	Fraction	Cutoff	IDs
New	Round 1	Phosphopeptides (Loc. prob. >0.75)	16255
New	Round 2	Phosphopeptides (Loc. prob. >0.75)	10928
Reuse	Round 1	Phosphopeptides (Loc. prob. >0.75)	16369
Reuse	Round 2	Phosphopeptides (Loc. prob. >0.75)	12812
New	Round 1	Peptides	5616
New	Round 2	Peptides	9803
Reuse	Round 1	Peptides	5175
Reuse	Round 2	Peptides	8656

Supplementary Table S8. Mean peptide and phosphopeptide (Loc. Prob. >0.75) IDs identified across three experimental replicates in a sequential enrichment approach with two rounds in which the beads were either exchanged after the first round or reused. The peptide input amount for the enrichment was 30 µg for all conditions.

Peptide input amount [µg]	Fraction	Cutoff	IDs
2.5	Round 1	Phosphopeptides (Loc. prob. >0.75)	8951
5	Round 1	Phosphopeptides (Loc. prob. >0.75)	11799
10	Round 1	Phosphopeptides (Loc. prob. >0.75)	13813
20	Round 1	Phosphopeptides (Loc. prob. >0.75)	15291
2.5	Round 2	Phosphopeptides (Loc. prob. >0.75)	3661
5	Round 2	Phosphopeptides (Loc. prob. >0.75)	6155
10	Round 2	Phosphopeptides (Loc. prob. >0.75)	9163
20	Round 2	Phosphopeptides (Loc. prob. >0.75)	12260
2.5	Round 3	Phosphopeptides (Loc. prob. >0.75)	1487
5	Round 3	Phosphopeptides (Loc. prob. >0.75)	2778
10	Round 3	Phosphopeptides (Loc. prob. >0.75)	5353
20	Round 3	Phosphopeptides (Loc. prob. >0.75)	8387
2.5	Round 4	Phosphopeptides (Loc. prob. >0.75)	841
5	Round 4	Phosphopeptides (Loc. prob. >0.75)	1631
10	Round 4	Phosphopeptides (Loc. prob. >0.75)	3296
20	Round 4	Phosphopeptides (Loc. prob. >0.75)	5800
2.5	Round 5	Phosphopeptides (Loc. prob. >0.75)	457
5	Round 5	Phosphopeptides (Loc. prob. >0.75)	922
10	Round 5	Phosphopeptides (Loc. prob. >0.75)	2027
20	Round 5	Phosphopeptides (Loc. prob. >0.75)	4085
2.5	Round 6	Phosphopeptides (Loc. prob. >0.75)	255
5	Round 6	Phosphopeptides (Loc. prob. >0.75)	507
10	Round 6	Phosphopeptides (Loc. prob. >0.75)	1244
20	Round 6	Phosphopeptides (Loc. prob. >0.75)	2105

Supplementary Table S9. Mean phosphopeptide IDs (Loc. prob. >0.75) identified across three experimental replicates using different peptide input amounts for a sequential six round enrichment. Each fraction (round) was obtained as eluate after the respective enrichment round and analyzed separately via LC-MS/MS.

Peptide input amount [µg]	Fraction	Cutoff	IDs
2.5	Pooled	Loc. prob. >0.75	7999
2.5	Cumulative	Loc. prob. >0.75	8776
2.5	Round 1	Loc. prob. >0.75	7814
2.5	Round 2	Loc. prob. >0.75	4858
5	Pooled	Loc. prob. >0.75	10511
5	Cumulative	Loc. prob. >0.75	10854
5	Round 1	Loc. prob. >0.75	9750
5	Round 2	Loc. prob. >0.75	6031
2.5	Pooled	3/3 valid values	6119
2.5	Cumulative	3/3 valid values	6786
2.5	Round 1	3/3 valid values	5956
2.5	Round 2	3/3 valid values	3667
5	Pooled	3/3 valid values	7979
5	Cumulative	3/3 valid values	8403
5	Round 1	3/3 valid values	7414
5	Round 2	3/3 valid values	4600

Supplementary Table S10. Mean phosphopeptide IDs with loc. prob. >0.75 or 3/3 valid intensity values among replicate intensity values identified across three experimental replicates using different peptide input amounts for a sequential two round enrichment. Each fraction (round) was either obtained as eluate after the respective enrichment round and analyzed separately via LC-MS/MS ("Round 1" and "Round 2") or obtained as a pooled eluate by reusing the elution buffer from the first enrichment round ("Pooled"). "Cumulative" refers to the cumulation of unique phosphopeptide IDs which were identified in the separate Fractions ("Round 1" & "Round 2") during data analysis.

Peptide input amount [µg]	Fraction	Cutoff	IDs
5	Normal	Phosphopeptides (Loc. prob. >0.75)	6737
5	Pooled	Phosphopeptides (Loc. prob. >0.75)	6385
5	Cumulative	Phosphopeptides (Loc. prob. >0.75)	5343
5	Round 1	Phosphopeptides (Loc. prob. >0.75)	4446
5	Round 2	Phosphopeptides (Loc. prob. >0.75)	3817
5	Round 3	Phosphopeptides (Loc. prob. >0.75)	2840
15	Normal	Phosphopeptides (Loc. prob. >0.75)	9247
15	Pooled	Phosphopeptides (Loc. prob. >0.75)	11356
15	Cumulative	Phosphopeptides (Loc. prob. >0.75)	10442
15	Round 1	Phosphopeptides (Loc. prob. >0.75)	8241
15	Round 2	Phosphopeptides (Loc. prob. >0.75)	7727
15	Round 3	Phosphopeptides (Loc. prob. >0.75)	6053
30	Normal	Phosphopeptides (Loc. prob. >0.75)	12568
30	Pooled	Phosphopeptides (Loc. prob. >0.75)	14085
30	Cumulative	Phosphopeptides (Loc. prob. >0.75)	13004
30	Round 1	Phosphopeptides (Loc. prob. >0.75)	10091
30	Round 2	Phosphopeptides (Loc. prob. >0.75)	8538
30	Round 3	Phosphopeptides (Loc. prob. >0.75)	8907

Supplementary Table S11. Mean phosphopeptide IDs (Loc. prob. >0.75) identified across three experimental using a three-round sequential enrichment approach with increasing Zr-IMAC HP bead volume for different peptide input amounts. “Normal” represents a standard single-round enrichment with 5 µL beads. “Pooled” represents a sequential enrichment for three rounds with increasing bead volume (Round 1: 1 µL beads, Round 2: +1 µL beads, Round 3: + 2 µL beads) and rounds pooled into the same elution buffer. “Round 1”, “Round 2” and “Round 3” represent the IDs identified in the respective separately collected and analyzed fractions. “Cumulative” refers to cumulation of unique phosphopeptide IDs identified in the separate fractions (“Round 1”, “Round 2”, “Round 3”) during data analysis.

Cell input	Mean peptide input [µg]	Cutoff	IDs
1 Mio.	59	Peptides	15262
500 k	34	Peptides	11105
200 k	15	Peptides	4978
100 k	8	Peptides	2026
50 k	3	Peptides	1573
10 k	1	Peptides	179
1 Mio.	59	Phosphopeptides	42665
500 k	34	Phosphopeptides	38585
200 k	15	Phosphopeptides	35653
100 k	8	Phosphopeptides	20666
50 k	3	Phosphopeptides	12558
10 k	1	Phosphopeptides	1147
1 Mio.	59	Phosphopeptides (Loc. prob. >0.75)	35490
500 k	34	Phosphopeptides (Loc. prob. >0.75)	32253
200 k	15	Phosphopeptides (Loc. prob. >0.75)	25206
100 k	8	Phosphopeptides (Loc. prob. >0.75)	14187
50 k	3	Phosphopeptides (Loc. prob. >0.75)	7967
10 k	1	Phosphopeptides (Loc. prob. >0.75)	711

Supplementary Table 12. Mean peptide, phosphopeptide and phosphopeptide (Loc. prob. >0.75) IDs identified across four experimental replicates on an Orbitrap Astral MS using different cell amounts within a HeLa dilution series. Mean peptide input amounts were determined *via* photometric measurement of peptide concentrations of all four experimental replicates.

Cell input	Mean peptide input [µg]	Cutoff	IDs
1 Mio.	59	Phosphosites	3190
500 k	34	Phosphosites	2910
200 k	15	Phosphosites	3294
100 k	8	Phosphosites	2129
50 k	3	Phosphosites	1518
10 k	1	Phosphosites	186
1 Mio.	59	Class I phosphosites (Loc. prob. >0.75)	30690
500 k	34	Class I phosphosites (Loc. prob. >0.75)	26779
200 k	15	Class I phosphosites (Loc. prob. >0.75)	21201
100 k	8	Class I phosphosites (Loc. prob. >0.75)	11708
50 k	3	Class I phosphosites (Loc. prob. >0.75)	6769
10 k	1	Class I phosphosites (Loc. prob. >0.75)	618

Supplementary Table 13. Mean phosphosite and class I phosphosite (Loc. prob. >0.75) IDs identified across four experimental replicates on an Orbitrap Astral MS using different cell amounts within a HeLa dilution series. Mean peptide input amounts were determined *via* photometric measurement of peptide concentrations of all four experimental replicates.
